# Structural and Mechanistic Basis for Extended-Spectrum Drug-Resistance Mutations in Altering the Specificity of TEM, CTX-M, and KPC β-lactamases

**DOI:** 10.3389/fmolb.2018.00016

**Published:** 2018-02-23

**Authors:** Timothy Palzkill

**Affiliations:** ^1^Department of Pharmacology and Chemical Biology, Baylor College of Medicine, Houston, TX, United States; ^2^Department of Biochemistry and Molecular Biology, Baylor College of Medicine, Houston, TX, United States

**Keywords:** β-lactamase, β-lactam antibiotics, protein evolution, antibiotic resistance, enzyme structure, enzyme mechanism

## Abstract

The most common mechanism of resistance to β-lactam antibiotics in Gram-negative bacteria is the production of β-lactamases that hydrolyze the drugs. Class A β-lactamases are serine active-site hydrolases that include the common TEM, CTX-M, and KPC enzymes. The TEM enzymes readily hydrolyze penicillins and older cephalosporins. Oxyimino-cephalosporins, such as cefotaxime and ceftazidime, however, are poor substrates for TEM-1 and were introduced, in part, to circumvent β-lactamase-mediated resistance. Nevertheless, the use of these antibiotics has lead to evolution of numerous variants of TEM with mutations that significantly increase the hydrolysis of the newer cephalosporins. The CTX-M enzymes emerged in the late 1980s and hydrolyze penicillins and older cephalosporins and derive their name from the ability to also hydrolyze cefotaxime. The CTX-M enzymes, however, do not efficiently hydrolyze ceftazidime. Variants of CTX-M enzymes, however, have evolved that exhibit increased hydrolysis of ceftazidime. Finally, the KPC enzyme emerged in the 1990s and is characterized by its broad specificity that includes penicillins, most cephalosporins, and carbapenems. The KPC enzyme, however, does not efficiently hydrolyze ceftazidime. As with the TEM and CTX-M enzymes, variants have recently evolved that extend the spectrum of KPC β-lactamase to include ceftazidime. This review discusses the structural and mechanistic basis for the expanded substrate specificity of each of these enzymes that result from natural mutations that confer oxyimino-cephalosporin resistance. For the TEM enzyme, extended-spectrum mutations act by establishing new interactions with the cephalosporin. These mutations increase the conformational heterogeneity of the active site to create sub-states that better accommodate the larger drugs. The mutations expanding the spectrum of CTX-M enzymes also affect the flexibility and conformation of the active site to accommodate ceftazidime. Although structural data are limited, extended-spectrum mutations in KPC may act by mediating new, direct interactions with substrate and/or altering conformations of the active site. In many cases, mutations that expand the substrate profile of these enzymes simultaneously decrease the thermodynamic stability. This leads to the emergence of additional global suppressor mutations that help correct the stability defects leading to increased protein expression and increased antibiotic resistance.

## Introduction

β-lactam antibiotics are the most often-used antimicrobials representing ~65% of antibiotic usage worldwide (Livermore, 2006). The β-lactams act by inhibiting bacterial cell wall biosynthesis. Specifically, they are covalent inhibitors of transpeptidase enzymes, commonly referred to as penicillin-binding proteins (PBPs). These enzymes catalyze a cross-linking reaction of pentapeptides present in the peptidoglycan layer of the cell wall (Lovering et al., 2012).

The most common mechanism of resistance to β-lactam antibiotics is the bacterial production of β-lactamases (Fisher et al., 2005). These enzymes catalyze the hydrolysis of the amide bond present in the β-lactam ring resulting in a product that is an ineffective inhibitor of the PBPs. β-lactamases can be distributed into four classes (A, B, C, and D) based on primary amino acid sequence homology (Ambler, 1980; Fisher et al., 2005). Classes A, C, and D are serine hydrolases that function similarly to classical serine proteases such as chymotrypsin. They catalyze attack of the catalytic serine on the carbonyl carbon of the amide bond to form a covalent acyl-enzyme intermediate that is subsequently hydrolyzed by a water molecule that has been activated by a general base. Class B β-lactamases are zinc metallo-enzymes that contain one or two zinc ions that coordinate a hydroxide ion for direct attack on the carbonyl carbon of the amide and do not proceed through a covalent acyl-enzyme (Palzkill, 2013).

Class A β-lactamases are often encoded on plasmids that can move by conjugation and, as a result, these enzymes are widespread sources of resistance (Bush and Fisher, 2011). Class A β-lactamases are a particular problem for resistance in Gram-negative bacteria including the ESKAPE pathogens (*Enterococcus faecium, Staphylococcus aureus, Klebsiella pneumonia, Acinetobacter baumannii, Pseudomonas aeruginosa*, and *Enterobacter* species; Pendleton et al., 2013). Individual class A β-lactamases display a range of substrate specificities although, as a group, they are commonly known for the efficient hydrolysis of penicillins and early generation cephalosporins. The extensive use of these antibiotics and subsequent spread of class A β-lactamases has led to widespread resistance (Bush and Fisher, 2011). This was countered in the 1980s with the introduction of oxyimino-cephalosporins, which are still good PBP inhibitors but poor substrates for β-lactamases. Mechanism-based inhibitors that target the β-lactamases were also developed to combat resistance (Drawz and Bonomo, 2010). The introduction and subsequent use of both these agents, however, placed selective pressure on bacteria resulting in the evolution of variants of class A enzymes that have gained the ability to hydrolyze oxyimino-cephalosporins or that avoid the action of β-lactamase inhibitors (Petrosino et al., 1998; Gniadkowski, 2008; Salverda et al., 2010).

**Figure 1:**
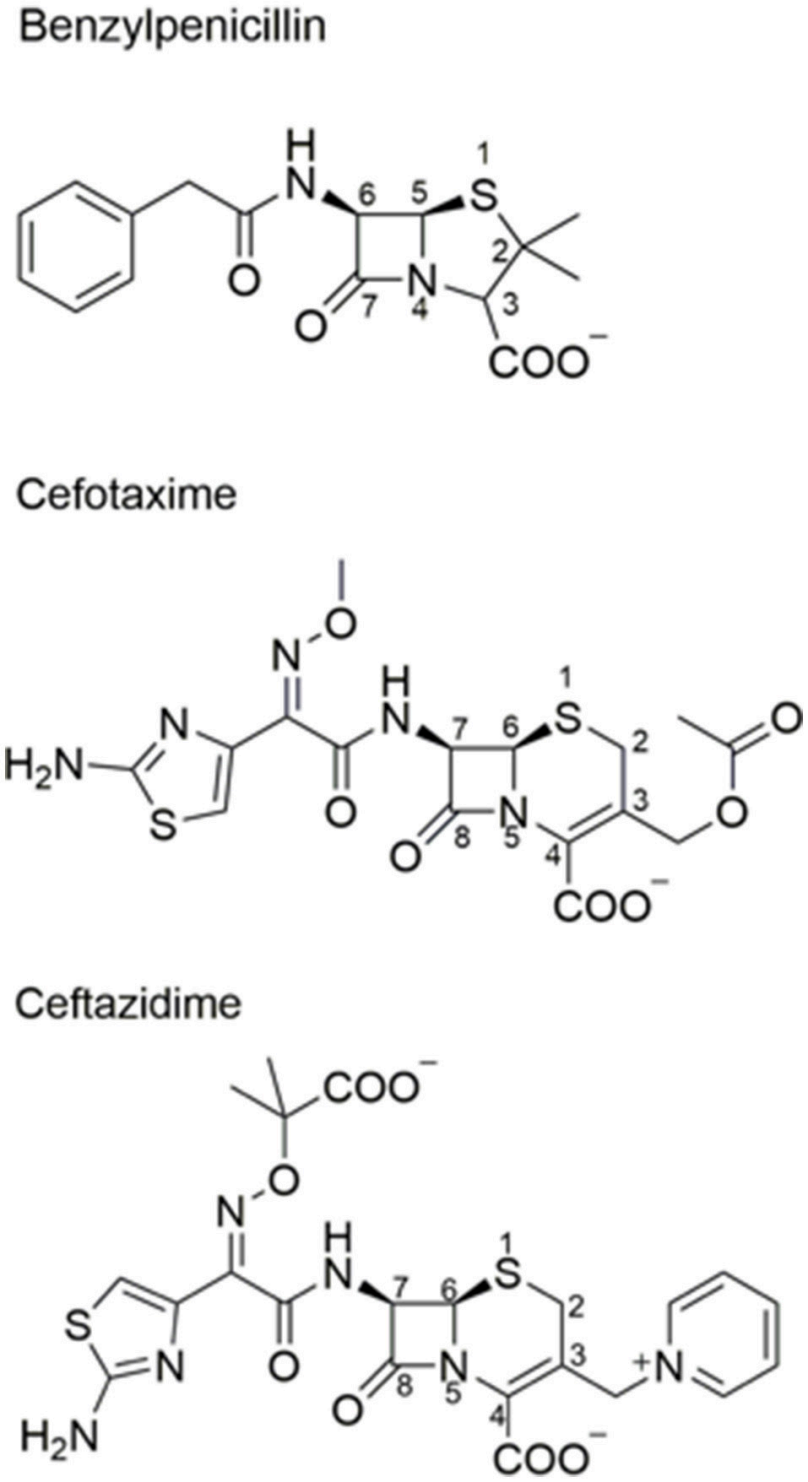
Chemical structures of β-lactam antibiotics. Benzylpenicillin and the oxyimino-cephalosporins cefotaxime and ceftazidime are shown.

This review will focus on three groups of class A β-lactamases that have evolved in response to the use of oxyimino-cephalosporins such as cefotaxime and ceftazidime and that are widespread sources of resistance in Gram-negative bacteria (Bonomo, 2017). These groups of enzymes include the TEM, CTX-M, and KPC β-lactamases. Although the evolution of resistance to mechanism-based inhibitors is clearly an important source of resistance, the focus of this review is on the evolution of enzymes with higher activity for catalysis of oxyimino-cephalosporins (Figure 1).

## Kinetics of β-lactam hydrolysis by class A β-lactamases

In order to understand the means by which the TEM, CTX-M, and KPC enzymes and variants inactivate oxyimino-cephalosporins, it is useful to review the kinetics and mechanism by which class A β-lactamases catalyze hydrolysis of β-lactams. Class A β-lactamases are serine hydrolases with a mechanism similar to serine proteases. After formation of the enzyme-substrate complex (ES), the active-site serine attacks and forms a covalent, acyl-enzyme intermediate (EAc). Subsequent catalysis of the hydrolysis of the acyl-enzyme generates the inactive, hydrolyzed β-lactamase product (P) (Hedstrom, 2002; Galleni and Frere, 2007).

$$E+S\underset{k_{-1}}{\overset{k_{1}}{⇄}}ES\overset{k_{2}}{\to }EAc\overset{k_{3}}{\to }E+P$$

Based on this mechanism, the Michaelis-Menten kinetic parameters are described by the following equations.

(1) $$\begin{array}{l} k_{cat}=k_{2}k_{3}/\left(k_{2}+k_{3}\right) \end{array}$$

(2) $$\begin{array}{l} K_{M}=k_{3}\left(k_{-1}+k_{2}\right)/k_{1}\left(k_{2}+k_{3}\right) \end{array}$$

(3) $$\begin{array}{l} k_{cat}/K_{M}=k_{1}k_{2}/\left(k_{-1}+k_{2}\right) \end{array}$$

For β-lactamases, the kinetic parameters *k*_cat_, *K*_M_, and *k*_cat_/*K*_M_ are composite constants that depend on the rates of multiple steps in the reaction. *k*_cat_ reflects the magnitude and relationship between the acylation (*k*_2_) and deacylation (*k*_3_) rate constants (Equation 1). *K*_M_ is often regarded as an indication of affinity between the substrate and enzyme, i.e., that *K*_M_ approximates *K*_s_, which is *k*_−1_/*k*_1_. However, in the β-lactamase mechanism, *K*_M_ is also dependent on *k*_2_ and *k*_3_ (Equation 2; Raquet et al., 1994). Assuming *k*_−1_>*k*_2_, *K*_M_ approximates substrate affinity (*K*_s_) when the acylation rate (*k*_2_) is much slower than the deacylation rate (*k*_3_), which is often not the case with β-lactam substrates. When deacylation is rate limiting (*k*_2_>*k*_3_), the value of *K*_M_ is lower than *K*_s_ and overestimates the affinity of the enzyme for substrate. Amino acid substitutions in β-lactamases that result in changes in *K*_M_ can be due to changes in *K*_s_, *k*_2_ or *k*_3_, or a combination thereof. Finally, *k*_cat_/*K*_M_ reflects the rates of steps occurring up to the formation of the acyl-enzyme. The deacylation rate (*k*_3_) does not contribute to the value of *k*_cat_/*K*_M_ as seen in equation 3.

## Class A β-lactamase mechanism

**Figure 2:**
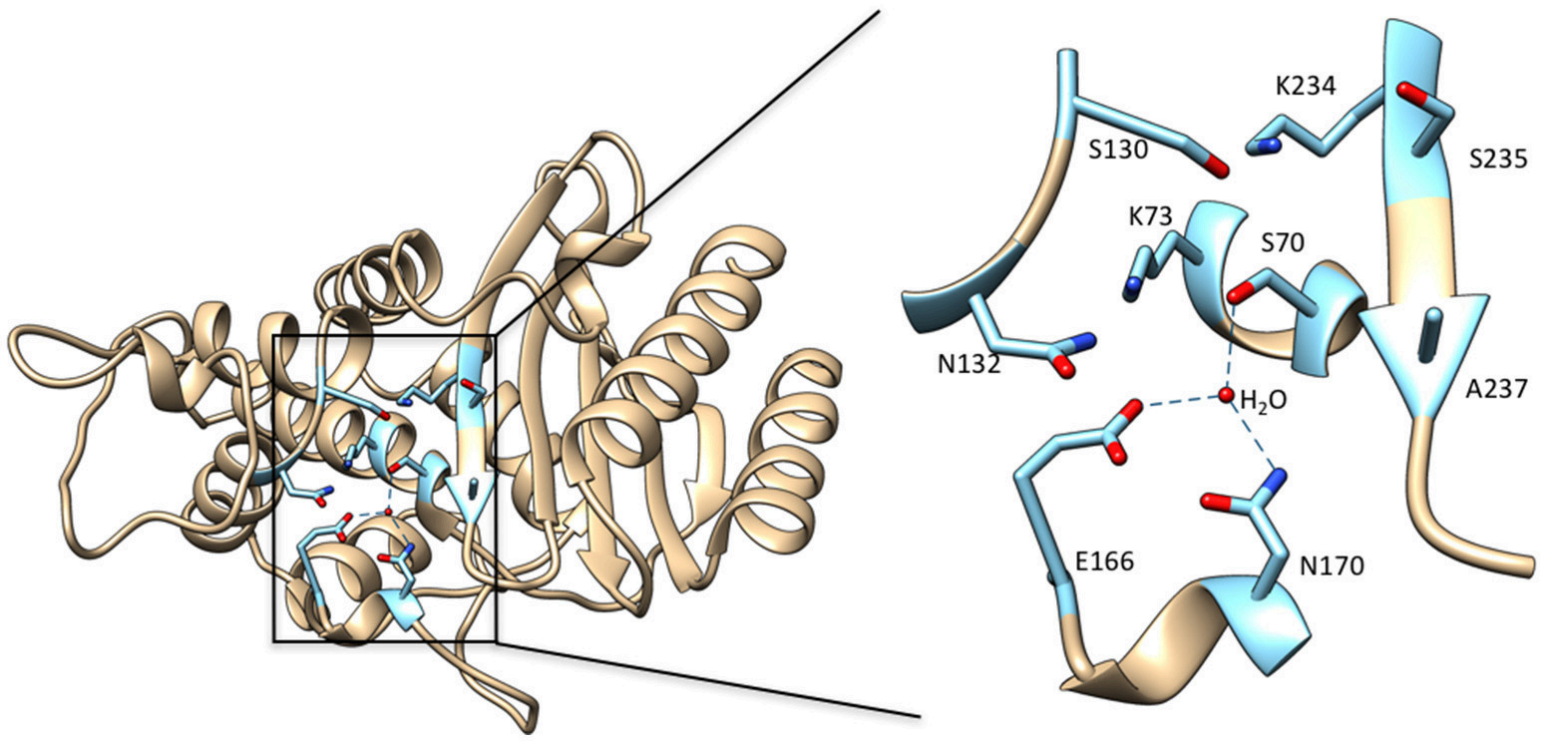
**Left**: Ribbon diagram of TEM-1 β-lactamase (PDB ID: 1BTL). Key active site residues that are implicated in substrate binding and catalysis and highlighted in cyan. **Right**: Enlarged view of the boxed region of the active site with the active site residues labeled. The deacylation water molecule is shown as a red sphere with hydrogen bonds from this water to Ser70, Glu166, and Asn170 shown as dotted lines. Hydrogen bonds between residues are not shown.

Class A β-lactamases utilize a set of conserved amino acid residues in the active site to promote substrate binding, acyl-enzyme formation, and subsequent deacylation to release product. The class A enzymes contain a β and an α/β domain, with the active site situated between these closely connected domains (Herzberg and Moult, 1987; Strynadka et al., 1992). Residues Ser70, Ser130, Asn132, Glu166, Asn170, Lys234, Ser/Thr235, Gly236, and Ala/Ser/Thr237 (Ambler numbering scheme; Ambler et al., 1991) are important contributors to substrate binding and catalysis (Figure 2). The hydroxyl oxygen of Ser70 serves as the nucleophile for attack on the carbonyl carbon of the amide bond (Strynadka et al., 1992; Fisher and Mobashery, 2009). The main chain NH groups of Ser70 and Ala237 act as the oxyanion hole and make hydrogen bonding interactions to stabilize the negative charge that develops on the carbonyl oxygen tetrahedral intermediate during acylation and deacylation (Strynadka et al., 1992). Although the details are controversial, Lys73, Glu166 and a catalytic water are thought to participate in abstracting the proton to activate Ser70 (Herzberg and Moult, 1987; Strynadka et al., 1992; Damblon et al., 1996; Minasov et al., 2002; Meroueh et al., 2005). Ser130 has been proposed to serve as a proton shuttle between Lys73 and the leaving group nitrogen (Strynadka et al., 1992). Asn132 forms a hydrogen bond with the acyl-amide group in the C6/7 side chain of penicillins and cephalosporins for binding and positioning of substrate. Lys234 and Ser/Thr235 make interactions with the C3/C4 substrate carboxylate found on all penicillins, cephalosporins and carbapenems and therefore are important for substrate binding. This interaction may also contribute to stabilization of the acylation and deacylation transition states to facilitate catalysis (Strynadka et al., 1992; Delmas et al., 2010; Fonseca et al., 2012). Glu166 serves as the general base for activating the catalytic water for the deacylation step and amino acid substitutions at this position lead to accumulation of the acyl-enzyme intermediate (Adachi et al., 1991; Delaire et al., 1991; Escobar et al., 1991; Strynadka et al., 1992). Asn170 forms a hydrogen bond to Glu166 and an active site water to help position the residue for activating the water. Both Glu166 and Asn170 are found on an omega loop structure that forms the base of the active site in class A β-lactamases (Figure 2). As discussed below, the omega loop plays an important role in the evolution of increased oxyimino-cephalosporin hydrolysis in class A enzymes. The residues described above are important for catalysis of all β-lactam substrates and mutations at these positions reduce activity. Thus, they form the core residues required for catalysis. Mutations leading to increased catalysis of oxyimino-cephalosporins occur at residues other than these core positions.

## TEM extended-spectrum β-lactamases (ESBLs)

TEM-1 β-lactamase was reported in 1963 in *E. coli* and *Salmonella* (Datta and Kontomichalou, 1965). It subsequently spread among Enterobacteriaceae and other Gram-negative pathogens to become a widespread source of β-lactam resistance. TEM-1 efficiently catalyzes the hydrolysis of penicillins and early generation cephalosporins and provides high-level bacterial resistance to these drugs. In part due to the widespread presence of TEM-1, the oxyimino-cephalosporins were introduced in the 1980s (Bush, 2002). The oximino-cephalosporins, such as cefotaxime and ceftazidime, include an oxyimino side chain at the C7 position (Figure 1). The large and inflexible oxyimino side chain is difficult to accommodate into the active site of TEM-1, resulting in slow rates of hydrolysis (Table 1). Ceftazidime differs from cefotaxime by having an even larger side chain that includes a carboxylate group (Figure 1). The increased bulk of ceftazidime is associated with a further reduction in rates of catalysis by TEM-1 compared to cefotaxime (Table 2).

**Table 1:** Kinetic parameters for cefotaxime hydrolysis by TEM-1 β-lactamase and mutants.

**Enzyme**	***k*_cat_ (s^−1^)**	**K_M_ (μM)**	***k*_cat_/K_M_ (M^−1^ s^−1^)**	**Buffer**	**References**
TEM-1 wt	0.25	450	5.6 × 10^2^	0.1 M phosphate, pH7.0, 25°C	Sowek et al., 1991
TEM-1 wt	9.0	6,000	1.5 × 10^3^	50 mM phosphate, pH7.0, 30°C	Raquet et al., 1994
TEM-1 wt	2	1,100	1.8 × 10^3^	pH7.0, 37°C	Petit et al., 1995
TEM-1 wt	2.5	1,684	1.5 × 10^3^	10 mM sodium bicarbonate, pH7.0, 37°C	Saves et al., 1995
TEM-1 wt	0.18	230	7.8 × 10^2^	50 mM phosphate, 100 mM NaCl, pH7.0, 25°C	Vakulenko et al., 1999
TEM-1 wt	nd^a^	nd	3.9 × 10^3^	50 mM phosphate, pH7.0, 30°C	Venkatachalam et al., 1994
TEM-1 wt	nd	nd	2.8 × 10^3^	50 mM phosphate, pH7.0, 30°C	Cantu and Palzkill, 1998
TEM-1 wt	0.64	308	2.1 × 10^3^	50 mM phosphate, pH7.0, 25°C	Wang et al., 2002
TEM-1 wt	nd	nd	2.1 × 10^3^	50 mM phosphate, pH7.0, 30°C	Brown et al., 2010
TEM-1 wt	nd	nd	1.0 × 10^3^	100 mM phosphate, pH7.0, 25°C	Dellus-Gur et al., 2015
TEM-1 wt	nd	nd	2.0 × 10^3^	50 mM phosphate, pH7.0, 10% glycerol, 25°C	Hart et al., 2016
TEM-1 wt	0.17	750	1.5 × 10^2^	100 mM phosphate, pH7.0, 25°C	Knies et al., 2017
Avg(SD)	2.1 (3.2)	1,500 (2,050)	1.7 × 10^3^ (1.0 × 10^3^)		
TEM R164S	2.4	230	1.0 × 10^4^	0.1 M phosphate, pH7.0, 25°C	Sowek et al., 1991
TEM R164S	0.2	174	1.1 × 10^3^	50 mM phosphate, 100 mM NaCl, pH7.0, 25°C	Vakulenko et al., 1999
TEM R164S	1.8	201	8.8 × 10^3^	50 mM phosphate, pH7.0, 25°C	Wang et al., 2002
TEM R164S	2.5	536	4.7 × 10^3^	100 mM phosphate, pH7.0, 25°C	Dellus-Gur et al., 2015
TEM R164S^b^	1.5	100	1.5 × 10^4^	50 mM phosphate, pH7.0, 30°C	Raquet et al., 1994
Avg(SD)	1.7 (1)	250 (170)	7.9 × 10^3^ (5.3 × 10^3^)		
TEM G238S	66	290	2.3 × 10^5^	50 mM phosphate, pH7.0, 30°C	Raquet et al., 1994
TEM G238S^b^	20	188	1.1 × 10^5^	10 mM sodium bicarbonate, pH7.0, 37°C	Saves et al., 1995
TEM G238S	7.5	577	1.3 × 10^4^	50 mM phosphate, pH7.0, 30°C	Viadiu et al., 1995
TEM G238S	16	124	1.4 × 10^5^	50 mM phosphate, pH7.0, 30°C	Cantu and Palzkill, 1998
TEM G238S	42	234	1.8 × 10^5^	50 mM phosphate, pH7.0, 25°C	Wang et al., 2002
TEM G238S	50	403	1.3 × 10^5^	100 mM phosphate, pH7.0, 25°C	Dellus-Gur et al., 2015
TEM G238S	50	190	2.6 × 10^5^	50 mM phosphate, pH7.0, 10% glycerol, 25°C	Hart et al., 2016
TEM G238S	14	700	2.2 × 10^4^	100 mM phosphate, pH7.0, 25°C	Knies et al., 2017
Avg(SD)	33 (21)	340 (200)	1.4 × 10^5^ (8.9 × 10^4^)		
TEM E104K	2.5	470	5.3 × 10^3^	0.1 M phosphate, pH7.0, 25°C	Sowek et al., 1991
TEM E104K	25	1,000	2.5 × 10^4^	pH7.0, 37°C	Petit et al., 1995
TEM E104K	9.3	980	9.4 × 10^3^	50 mM phosphate, pH7.0, 25°C	Wang et al., 2002
TEM E104K	nd	nd	1.2 × 10^4^	50 mM phosphate, pH7.0, 10% glycerol, 25°C	Hart et al., 2016
TEM E104K	3.9	5,000	6.0 × 10^2^	100 mM phosphate, pH7.0, 25°C	Knies et al., 2017
Avg(SD)	10 (10)	1,870 (2,110)	1.0 × 10^4^ (9.2 × 10^3^)		
TEM E240K	0.66	140	4.7 × 10^3^	0.1 M phosphate, pH7.0, 25°C	Sowek et al., 1991
TEM E240K	nd	nd	8.5 × 10^3^	50 mM phosphate, pH7.0, 30°C	Venkatachalam et al., 1994
Avg(SD)	–	–	6.6 × 10^3^ (2.7 × 10^3^)		

a *nd, not determined*.

b *Enzyme also contains Q39K substitution*.

**Table 2:** Kinetic parameters for ceftazidime hydrolysis by TEM-1 β-lactamase and mutants.

**Enzyme**	***k*_cat_ (s^−1^)**	**K_M_ (μM)**	***k*_cat_/K_M_ (M^−1^s^−1^)**	**Buffer**	**References**
TEM-1 wt	0.0016	80	20	0.1 M phosphate, pH7.0, 25°C	Sowek et al., 1991
TEM-1 wt	0.3	4,300	70	50 mM phosphate, pH7.0, 30°C	Raquet et al., 1994
TEM-1 wt	nd^a^	nd	21	50 mM phosphate, pH7.0, 30°C	Venkatachalam et al., 1994
TEM-1 wt	0.02	300	66	pH7.0, 37°C	Petit et al., 1995
TEM-1 wt	nd	nd	55	50 mM phosphate, pH7.0, 30°C	Cantu et al., 1996
TEM-1 wt	0.008	200	40	50 mM phosphate, 100 mM NaCl, pH7.0, 25°C	Vakulenko et al., 1999
TEM-1 wt	0.018	557	32	50 mM phosphate, pH7.0, 25°C	Wang et al., 2002
Avg(SD)	0.07 (0.13)	1,090 (1,800)	43 (21)		
TEM R164S	3.4	260	1.3 × 10^4^	0.1 M phosphate, pH7.0, 25°C	Sowek et al., 1991
TEM R164S^b^	9	1,000	9.0 × 10^3^	50 mM phosphate, pH7.0, 30°C	Raquet et al., 1994
TEM R164S	1.4	270	5.2 × 10^3^	50 mM phosphate, 100 mM NaCl, pH7.0, 25°C	Vakulenko et al., 1999
TEM R164S	8.5	1,600	5.3 × 10^3^	50 mM phosphate, pH7.0, 25°C	Wang et al., 2002
Avg(SD)	5.6 (3.8)	780 (650)	8.1 × 10^3^ (3.7 × 10^3^)		
TEM G238S	0.9	532	1.6 × 10^3^	50 mM phosphate, pH7.0, 30°C	Venkatachalam et al., 1994
TEM G238S	26	5,200	5.0 × 10^3^	50 mM phosphate, pH7.0, 30°C	Raquet et al., 1994
TEM G238S	0.55	897	6.1 × 10^2^	50 mM phosphate, pH7.0, 25°C	Wang et al., 2002
TEM G238S	1.0	343	3.0 × 10^3^	50 mM phosphate, pH7.0, 30°C	Cantu and Palzkill, 1998
Avg(SD)	7 (13)	1,740 (2,320)	2.6 × 10^3^ (1.9 × 10^3^)		
TEM E104K	0.07	150	4.5 × 10^2^	0.1 M phosphate, pH7.0, 25°C	Sowek et al., 1991
TEM E104K	0.3	80	3.7 × 10^3^	pH7.0, 37°C	Petit et al., 1995
TEM E104K	0.41	760	5.4 × 10^2^	50 mM phosphate, pH7.0, 25°C	Wang et al., 2002
Avg(SD)	0.26 (0.17)	330 (370)	1.6 × 10^3^ (1.9 × 10^3^)		
TEM E240K	0.28	460	6.1 × 10^2^	0.1 M phosphate, pH7.0, 25°C	Sowek et al., 1991
TEM E240K	nd	nd	1.7 × 10^3^	50 mM phosphate, pH7.0, 30°C	Venkatachalam et al., 1994
Avg(SD)	–	–	1.2 × 10^3^ (7.7 × 10^2^)		

a *nd, not determined*.

b *Enzyme also contains Q39K substitution*.

The kinetic parameters for oxyimino-cephalosporin hydrolysis by TEM-1 reveal low *k*_cat_ and *k*_cat_/*K*_M_ values and high *K*_M_ values compared with good substrates such as ampicillin or benzylpenicillin. There have been a large number of studies on cefotaxime hydrolysis by TEM-1 and a list of kinetic parameters is compiled in Table 1. The list is likely not comprehensive and there is some variation in assay conditions such as 25°C vs. 30°C but the majority of the listed results were obtained using similar buffer conditions. There is some variation in published values, particularly with regard to *K*_M_. *K*_M_ is generally high and in several studies it was too high to measure accurately (Table 1). The *k*_cat_/*K*_M_ values, however, are relatively consistent and average ~1.7 × 10^3^ M^−1^s^−1^ (Table 1). Published kinetic parameters for ceftazidime hydrolysis also exhibit variation in *K*_M_ but with *K*_M_ generally high (Table 2). However, *k*_cat_/*K*_M_ values are quite consistent and average ~40 M^−1^s^−1^. Therefore, both cefotaxime and ceftazidime are poor substrates for TEM-1 β-lactamase. Ceftazidime, however, is a particularly poor substrate with an ~45-fold lower *k*_cat_/*K*_M_ value than that observed for cefotaxime hydrolysis. Published *k*_cat_/*K*_M_ values for benzylpenicillin hydrolysis are in the range of 10^7^-10^8^ M^−1^s^−1^ and therefore cefotaxime and ceftazidime hydrolysis is 10^4^-10^6^-fold less efficient (Christensen et al., 1990). Since *k*_cat_/*K*_M_ reflects the events occurring up to formation of the acyl-enzyme, the low *k*_cat_/*K*_M_ values for cefotaxime and ceftazidime hydrolysis reflect low affinity for substrate binding and/or a slow acylation reaction. Indeed, estimates of *K*_s_ for the binding of cefotaxime and ceftazidime to TEM-1 reveal poor affinity with values of 3.8 and 9.9 mM, respectively (Vakulenko et al., 1999). The rate-limiting step for cefotaxime hydrolysis by TEM-1 has been shown to be the acylation step (*k*_2_) (Saves et al., 1995). Taken together, these results are consistent with the large, inflexible oxyimino side chain of cefotaxime being poorly accommodated in the active site of TEM-1.

**Figure 3:**
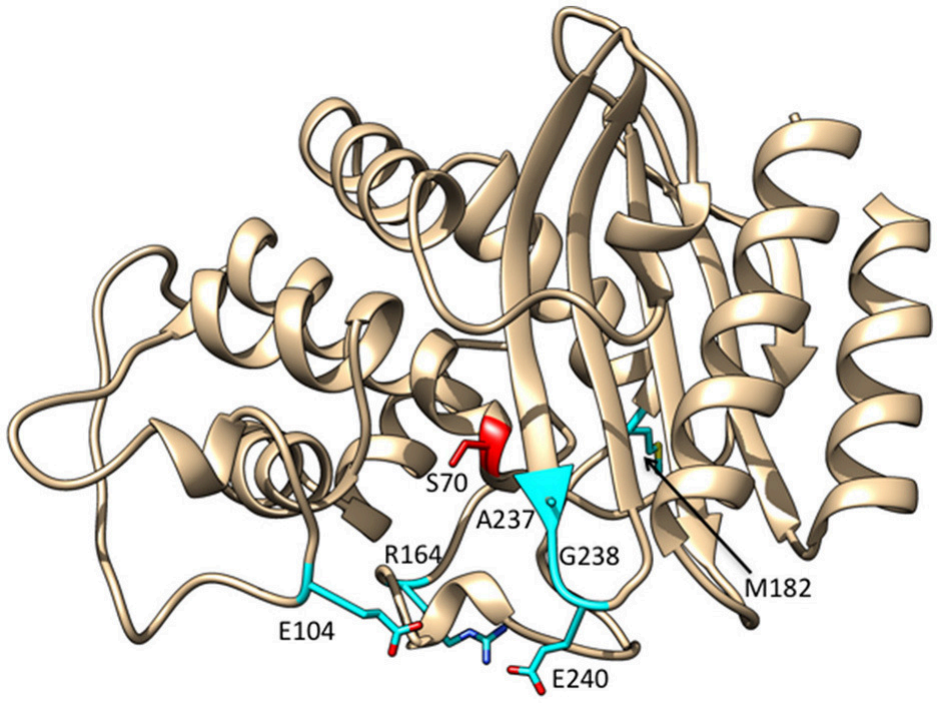
Ribbon diagram of TEM-1 β-lactamase showing the positions at which substitutions commonly occur among enzyme variants with increased catalytic activity for oxyimino-cephalosporins highlighted in cyan. The active site Ser70 nucleophile is highlighted in red.

Since the introduction of oxyimino-cephalosporins into clinical practice, variants of TEM-1 with amino acid substitutions that result in increased hydrolysis have been emerging (Petrosino et al., 1998; Salverda et al., 2010; Pimenta et al., 2014). Each unique variant is given a new number and these variants are termed TEM extended spectrum β-lactamases (ESBLs). The number of TEM variants is now greater than 200 (Salverda et al., 2010; Pimenta et al., 2014). The most common substitutions in TEM enzymes displaying enhanced oxyimino-cephalosporin hydrolysis include E104K, R164S/H, M182T, A237T, G238S, and E240K (Figure 3). The effects of these substitutions have been studied extensively using kinetic, biophysical, and structural tools to determine the mechanism by which resistance to oxyimino-cephalosporins is evolving.

## G238S and R164S substitutions

The R164S and G238S substitutions are associated with the largest increases in cefotaxime and ceftazidime hydrolysis when introduced into the TEM-1 enzyme. These substitutions are likely the driver substitutions for clinically relevant resistance and are the most often observed in TEM ESBLs. The G238S substitution is predominantly associated with enhanced hydrolysis of cefotaxime and increased resistance. Residue 238 is situated on the β3 strand that forms a side of the active site (Figure 3). A number of studies have shown that the G238S substitution, when introduced into the TEM-1 enzyme, results in an ~80-fold increase in *k*_cat_/*K*_M_ for cefotaxime hydrolysis compared to wild-type TEM-1 and has a value of ~1.4 × 10^5^ M^−1^s^−1^ (Table 1). It is somewhat difficult to estimate the changes in *k*_cat_ and *K*_M_ relative to TEM-1 as they are often not determined for wild type because of high *K*_M_ values. However, it is clear that *K*_M_ for cefotaxime hydrolysis is reduced for G238S compared to TEM-1 as the value is consistently measurable and is in the range of 300 μM while *k*_cat_ is in the range of 30 s^−1^ (Table 1). Saves et al. utilized electrospray mass spectroscopy to determine acylation (*k*_2_) and deacylation (*k*_3_) rates for TEM-1 and the G238S mutant and found acylation is rate-limiting for cefotaxime hydrolysis for TEM-1, as noted above (Saves et al., 1995). Acylation remains rate-limiting for the G238S enzyme but *k*_2_ is increased nearly 10-fold (Saves et al., 1995). A significant portion of the increase in *k*_cat_/*K*_M_ observed for the G238S enzyme is thus due to an increase in the acylation rate. Although values for *K*_s_ for G238S with cefotaxime have not been determined, the fact that acylation is rate-limiting indicates *K*_M_ is an approximation of *K*_s_. Since the *K*_M_ for cefotaxime is reduced for the G238S enzyme compared to TEM-1 (Table 1), this suggests that the increased affinity of the G238S enzyme for cefotaxime also contributes to the increase in *k*_cat_/*K*_M_.

The G238S substitution in TEM-1 also results in increased ceftazidime hydrolysis, albeit to a lesser extent than that observed for cefotaxime, with a *k*_cat_/*K*_M_ value of ~2.5 × 10^3^ M^−1^s^−1^ (Table 2). This is a 60-fold increase over that observed for TEM-1. As with cefotaxime, *k*_cat_ and *K*_M_ comparisons to wild-type TEM-1 are difficult. However, the *K*_M_ for ceftazidime hydrolysis is reduced to a measurable range for G238S with a value of ~1,700 μM while *k*_cat_ is roughly 7 s^−1^, although there is high variance in published values (Table 2). A comparison of *k*_cat_/*K*_M_ values for G238S reveals a 60-fold higher catalytic efficiency for cefotaxime vs. ceftazidime hydrolysis.

While the G238S substitution increases the rate of cefotaxime and ceftazidime hydrolysis, it significantly decreases the rate of hydrolysis of penicillins. For example, *k*_cat_/*K*_M_ for ampicillin hydrolysis is reduced 10-fold compared to wild-type TEM-1 while *k*_cat_ is decreased ~50-fold (Dellus-Gur et al., 2015). Very similar effects were observed for hydrolysis of benzylpenicillin by the G238S enzyme (Cantu and Palzkill, 1998). Because *k*_cat_ is decreased, the effect of G238S is not simply on binding affinity for penicillins but includes reduced rates of acylation (*k*_2_), deacylation (*k*_3_), or both. Christensen et al. determined that, for TEM-1, the rates of acylation and deacylation are fast (>1,000 s^−1^) and neither is rate-limiting for benzylpenicillin hydrolysis (Christensen et al., 1990). Using mass spectroscopy, Saves et al. determined that, for the G238S enzyme, *k*_3_ is greatly reduced and is rate-limiting for hydrolysis of benzylpenicillin (Saves et al., 1995). Based on these results, it was suggested the G238S substitution may affect the positioning of the omega loop and alter the action of the Glu166 general base, which is present on the loop, resulting in a decreased deacylation rate (Saves et al., 1995).

Two models have been proposed to explain the mechanism of the G238S substitution. One model involves a hydrogen bond between the side chain hydroxyl of the Ser238 residue and the oxime group of the oxyimino-cephalosporins that would improve affinity of the enzyme for the antibiotics and thereby increase catalytic efficiency (Huletsky et al., 1993). The second model suggests that steric conflicts of the Ser238 side chain with residue Asn170 would lead to movement of the β3 strand or movement of the omega loop thereby causing expansion of the active site to accommodate the larger oxyimino-cephalosporin side chain (Saves et al., 1995; Cantu and Palzkill, 1998). An analysis of kinetic parameters of a series of substitutions at position 238 supported the steric conflict model in that *k*_cat_/*K*_M_ for oxyimino-cephalosporin hydrolysis correlated more closely with side-chain volume than hydrogen bonding potential (Cantu and Palzkill, 1998). In addition, docking and molecular dynamics studies of the G238S mutant with cefotaxime have suggested Ser238 does not hydrogen bond to cefotaxime in the complex (Singh and Dominy, 2012).

**Figure 4:**
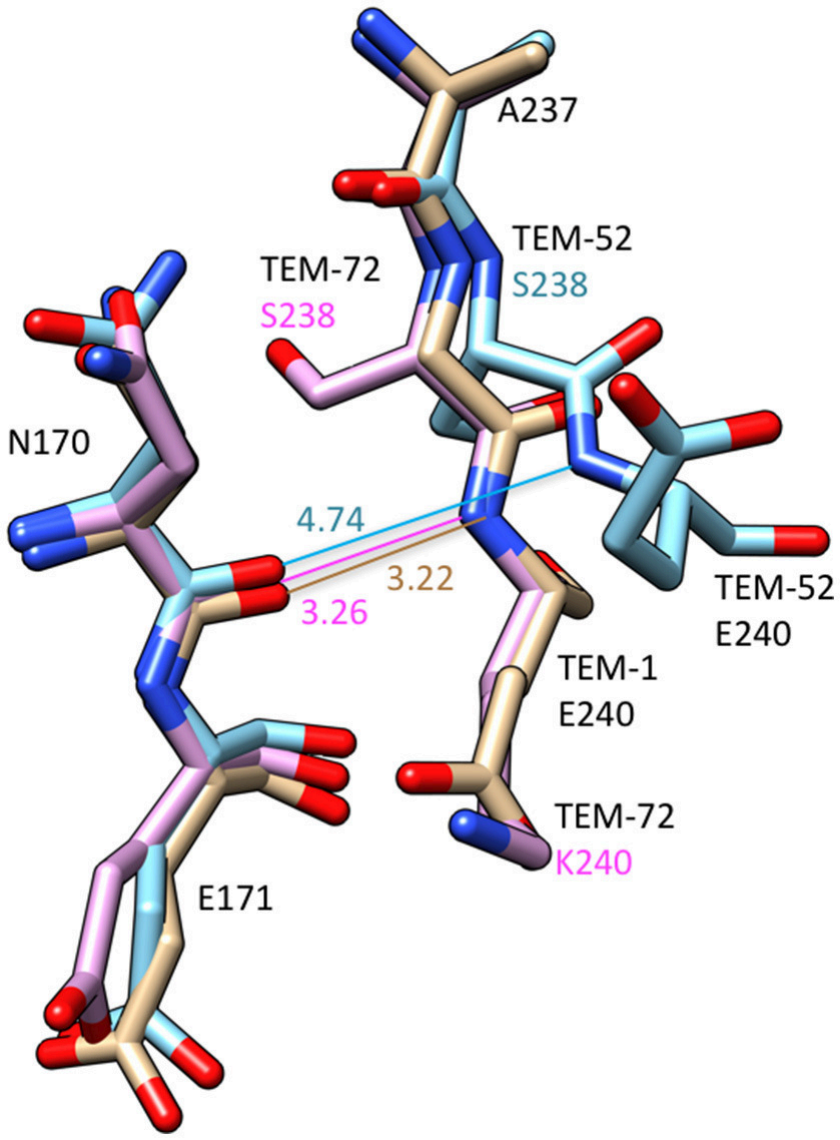
Schematic illustration of a structural comparison of TEM-1 (tan) (PDB ID: 1BTL), TEM-52 (cyan) (1HTZ), and TEM-72 (pink) (3P98). The omega loop residues Asn170-Glu171 and the end of the β3-sheet containing positions 237–240 are shown. The distance between the Asn170 carbonyl oxygen and residue 240 main chain NH are shown. The numbers indicate distances in Å. The distance lines are color coded to match the molecules. The G238S substitution in TEM-52 is associated with a movement of residue 238 and 240 away from Asn170 creating additional space in the active site. Labels for amino acid residues that are substituted relative to wild type are colored according to structure with TEM-52 (cyan), TEM-72 (pink).

The structures of TEM variants from clinical isolates containing multiple substitutions including G238S have been determined. The structure of the TEM-52 enzyme containing the E104K/M182T/G238S substitutions shows the position of the loop region of residues 238–243 is moved by 2.8 Å to widen the active site to accommodate the bulkier side chains of oxyimino-cephalosporins (Figure 4). It was also noted that lysine from the E104K substitution is oriented toward the active site (Orencia et al., 2001). In addition, the structure of the TEM-72 enzyme containing the Q39K, M182T, G238S, and E240K substitutions has been determined (Docquier et al., 2011). Curiously, the TEM-72 enzyme does not show the movement of the 238–243 loop and the active site is not expanded (Figure 4). This could be due to the presence of the E240K substitution adjacent to G238S somehow restraining the conformation. Note, however, that TEM-1 containing the G238S/E240K double mutant has higher catalytic efficiency for cefotaxime hydrolysis than G238S alone, indicating cefotaxime is accommodated into the active site in the simultaneous presence of G238S and E240K (Venkatachalam et al., 1994). Finally, the structure of a TEM G238A mutant has been determined and, similar to TEM-52, shows an expansion of the active site, however, the expansion is due to movement of the omega loop because of steric conflict with the substituted alanine (Wang et al., 2002).

The X-ray crystal structure of the G238S mutant has also been determined in a TEM-1 enzyme that also contains stabilizing substitutions that enhance protein expression and crystallization (Dellus-Gur et al., 2015). These substitutions did not influence the catalytic properties of the enzyme compared to G238S without the stabilizing substitutions. The G238S structures were determined under cryogenic conditions and at room temperature (Dellus-Gur et al., 2015). The G238S enzyme exists in two well-defined conformations of the active site loop at the end of the β3-strand containing residue 238. One of these is a more open conformation that could accommodate cefotaxime (Dellus-Gur et al., 2015). Taken together, the structural results on G238S suggest, TEM-72 notwithstanding, the substitution is associated with increased space to accommodate substrate and are broadly consistent with the steric conflict model.

The R164S substitution results in increased hydrolysis of both cefotaxime and ceftazidime, although it is most often associated with TEM ESBLs providing increased resistance to ceftazidime. Several studies reveal the R164S substitution results in a roughly 5-fold increase in *k*_cat_/*K*_M_ for cefotaxime hydrolysis compared to TEM-1 with a value of ~ 8.0 × 10^3^ M^−1^s^−1^ (Table 1). Although cefotaxime *K*_M_ values are often too high to measure for TEM-1, *K*_M_ is measureable for R164S with values in the range of 250 μM and *k*_cat_ in the range of 2 s^−1^ (Table 1). In contrast to the relatively modest increase in cefotaxime hydrolysis mediated by R164S, this substitution is associated with an ~200-fold increase in *k*_cat_/*K*_M_ for ceftazidime hydrolysis compared to TEM-1 with a value of ~8.0 × 10^3^ M^−1^s^−1^ compared to 40 M^−1^s^−1^ for TEM-1 (Table 2). The increased catalytic efficiency is associated with a consistently measurable *K*_M_ for R164S with a value of ~800 μM and a *k*_cat_ of ~6 s^−1^. The large increase in *k*_cat_/*K*_M_ for ceftazidime hydrolysis by R164S relative to TEM-1 may explain why the substitution is often associated with ESBLs with high ceftazidime resistance. Because *k*_cat_/*K*_M_ is a reflection of rates occurring up to the formation of the acyl-enzyme, the increase in *k*_cat_/*K*_M_ relative to TEM-1 for both cefotaxime and ceftazidime hydrolysis by R164S suggests the substitution is associated with a decrease in *K*_s_ (increased affinity) and/or an increase in *k*_2_.

As with G238S, the R164S substitution is associated with decreased penicillin hydrolysis. For example, the *k*_cat_ for ampicillin hydrolysis is decreased 25-fold and *k*_cat_/*K*_M_ is decreased 13-fold for the R164S enzyme relative to wild-type TEM-1 (Dellus-Gur et al., 2015). Similarly, for benzylpenicillin hydrolysis, *k*_cat_ is decreased 70-fold and *k*_cat_/*K*_M_ is decreased 14-fold (Vakulenko et al., 1999). There are no reported values for *K*_s_, *k*_2_ or *k*_3_ for penicillin hydrolysis but the 25- and 70-fold reductions in *k*_cat_ suggests *k*_2_ and/or *k*_3_ values are significantly lower for penicillin hydrolysis by R164S relative to TEM-1.

**Figure 5:**
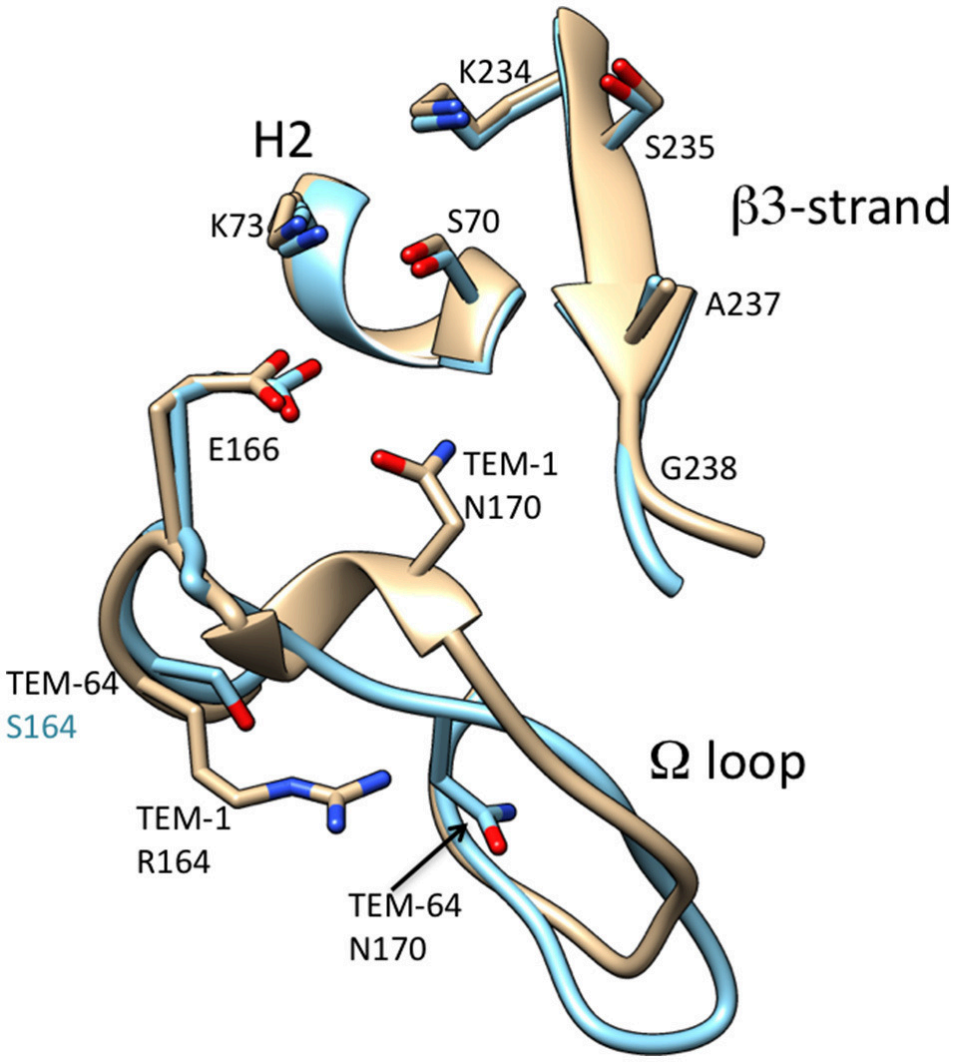
Schematic illustration of the omega loop region as well as the β3-strand and H2 helix of the active site of TEM-1 (tan) (PDB ID: 1BTL) in comparison to that of TEM-64 (cyan) (1JWZ). The omega loop in TEM-64 undergoes a large conformational change moving Asn170 out of the active site. Labels for amino acid residues that are substituted relative to wild type are colored according to structure with TEM-64 (cyan). The boronic acid inhibitor of the TEM-64 structure is omitted for clarity.

Several groups have investigated the structural basis of the change in substrate specificity provided by R164S. Mobashery and colleagues noted that the R164S substitution would create a cavity in the middle of the omega loop and the top of the loop containing Pro167 and Asn170 would collapse to fill the void. This rearrangement creates additional space in the active site (Vakulenko et al., 1999). The structure of TEM-64 (E104K/R164S/M182T), which contains the R164S substitution, has been determined (Wang et al., 2002). It was found the omega loop has an altered conformation where the short helix in the region of residue 170 unwinds and Asn170 moves by 4.5 Å to create a cavity in the active site that could better accommodate the C7β side chain of oxyimino-cephalosporins (Wang et al., 2002; Figure 5). The structure of the R164S mutant has also been determined in a TEM-1 enzyme that, like G238S described above, also contains stabilizing substitutions that enhance protein expression and crystallization without affecting kinetic parameters (Dellus-Gur et al., 2015). Similar to G238S described above, structures were determined at cryogenic conditions and room temperature. The structure of R164S showed the omega loop is conformationally heterogenous (Dellus-Gur et al., 2015). An ensemble of different conformations of the omega loop are present, some of which better accommodate cefotaxime. Binding of oxyimino-cephalosporins is proposed to shift the ensemble toward catalytically active conformations. The structure of a covalently bound boronic-acid analog showed less conformational heterogeneity, supporting this view (Dellus-Gur et al., 2015).

## E104K and E240K substitutions

The E104K and E240K substitutions are also associated with increases in the hydrolysis of cefotaxime and ceftazidime. Although these substitutions have been found individually in ESBLs, they are often found in association with the G238S or R164S substitutions. The E104K substitution has been studied by several groups and exhibits an ~6-fold increase in *k*_cat_/*K*_M_ for cefotaxime hydrolysis compared to TEM-1 with a value of 1.0 × 10^4^ M^−1^s^−1^(Table 1). *K*_M_ is consistently measurable for TEM-1 containing the E104K substitution with an average of 1,800 μM, although with high variance in values. *k*_cat_ is likely also increased relative to TEM-1 and has a value of ~10 s^−1^ (Table 1). For ceftazidime hydrolysis, the E104K substitution is associated with an ~40-fold increase in *k*_cat_/*K*_M_ compared to TEM-1 (Table 2). The *K*_M_ value for E104K with ceftazidime is in a measurable range with a value of ~300 μM while *k*_cat_ is ~0.3 s^−1^ (Table 2). There are no published results on *K*_s_, *k*_2_ or *k*_3_ for E104K for these substrates but the increased *k*_cat_/*K*_M_ values suggest minimally that *K*_s_ is reduced and/or *k*_2_ is increased by the substitution. It has been suggested that electrostatic interactions of E104K could increase binding of oxyimino-cephalosporins. Docking and molecular dynamics, however, suggest the lysine at position 104 does not directly interact with bound cefotaxime, although ceftazidime was not examined (Sowek et al., 1991; Singh and Dominy, 2012).

In contrast to the R164S and G238S substitutions, the E104K substitution has little effect on the hydrolysis of penicillins. The kinetic parameters for benzylpenicillin hydrolysis are very similar to TEM-1 with high *k*_cat_, low *K*_M_ and very high *k*_cat_/*K*_M_ values (Sowek et al., 1991; Hart et al., 2016). Similarly, kinetic parameters are largely unchanged for hydrolysis of 6-furylacrylpenicillanic acid (FAP) (Wang et al., 2002). These results suggest the E104K substitution does not significantly modify active site structures that are necessary for penicillin hydrolysis. Substitutions in TEM-1 that increase oxyimino-cephalosporin hydrolysis therefore need not reduce hydrolysis rates of excellent substrates such as penicillins.

The E240K substitution has been evaluated in multiple publications and exhibits an ~4-fold increase in *k*_cat_/*K*_M_ for cefotaxime hydrolysis compared to TEM-1 with a value of ~6.6 × 10^3^ M^−1^s^−1^ (Table 1). The *K*_M_ for cefotaxime hydrolysis is decreased to a measurable value of ~140 μM while *k*_cat_ is low and has a value of ~0.5 s^−1^. With regard to ceftazidime hydrolysis, the E240K substitution results in a roughly 30-fold increase in *k*_cat_/*K*_M_ compared to TEM-1 (Table 2). Similar to the E104K substitution, the E240K substitution results in only modest changes in kinetic parameters for hydrolysis of penicillins such as benzylpenicillin (Sowek et al., 1991). Thus, like E104K, the E240K enzyme is an excellent penicillinase. Finally, a comparison of the effects of E104K vs. E240K on *k*_cat_/*K*_M_ for cefotaxime and ceftazidime reveals that both substitutions result in a ~5-fold increase for cefotaxime but a 30–40-fold increase for ceftazidime relative to TEM-1, indicating these substitutions have the largest impact on ceftazidime hydrolysis.

## A237T substitution

The A237T substitution occurs more rarely than R164S or G238S among TEM ESBLs and when it occurs, it is associated with other substitutions, particularly R164S (www.lahey.org/studies/webt.asp). The A237T substitution was first identified in a selection for mutants of TEM-1 with increased cephalosporin C resistance (Hall and Knowles, 1976). There have been relatively few detailed biochemical studies of the effect of the A237T substitution. Healy et al. showed with purified enzyme that the A237T substitution results in a 2-fold decrease in *k*_cat_ but a modest (1.3-fold) increase in *k*_cat_/*K*_M_ for hydrolysis of the cephalosporins cephalothin and cephalosporin C (Healy et al., 1989). In addition, they showed the substitution reduces hydrolysis of benzylpenicillin and ampicillin with a 7-fold decrease in *k*_cat_ and a ~10-fold decrease in *k*_cat_/*K*_M_. The A237T substitution also decreases the thermal stability of the enzyme by 5°C. Further, Cantu et al. made similar observations with regard to the negative effect of A237T on benzylpenicillin and ampicillin hydrolysis and also showed the substitution causes a modest 1.3-fold increase in *k*_cat_/*K*_M_ for cefotaxime (Cantu et al., 1997). A further suggestion that the A237T substitution increases cefotaxime hydrolysis comes from a study showing that TEM-24 (Q39K/E104K/R164S/A237T/E240K) exhibits a ~10-fold higher V_max_/*K*_M_ value for cefotaxime hydrolysis relative to TEM-46 (Q39K/E104K/R164S/E240K) (Chanal-Claris et al., 1997). There is no structural information available on a TEM A237T mutant; however, by analogy to structures of the class A enzymes Toho-1, CTX-M-9, and CTX-M-14 in complex with cefotaxime, it is possible that the hydroxyl group of Thr237 makes a hydrogen bond to the C4 carboxylate group to enhance cefotaxime hydrolysis (Shimamura et al., 2002; Delmas et al., 2010; Adamski et al., 2015).

## M182T substitution

The M182T substitution is found in many TEM ESBL enzymes as well as inhibitor resistant enzymes. M182T is found in combination with other amino acid substitutions in ESBLs (www.lahey.org/studies/webt.asp). In contrast to the substitutions described above, residue 182 is not in the vicinity of the active site (Figure 3). The M182T substitution, when introduced by itself into the TEM-1 enzyme, does not alter substrate specificity (Sideraki et al., 2001; Wang et al., 2002). Instead, M182T acts by increasing thermodynamic stability and suppressing aggregation (Sideraki et al., 2001; Wang et al., 2002). A role for the M182T substitution in ESBL evolution was found in a genetic study to identify intragenic second-site suppressor mutations of folding and stability mutants in TEM-1 β-lactamase (Huang and Palzkill, 1997). For this purpose, a destabilizing mutation in the hydrophobic core (L76N) was introduced into TEM-1. This resulted in rapid proteolysis of the enzyme in *E. coli* and a large reduction in ampicillin resistance. Suppressor mutations (i.e., mutations that restored ampicillin resistance to *E. coli* containing the TEM-1 L76N gene) were selected after random introduction of point mutations in the TEM gene (Huang and Palzkill, 1997). Using this approach, mutants that reverted the L76N substitution to leucine and isoleucine were discovered; however, the most common suppressor was M182T, which is located 17Å from residue 76 (Huang and Palzkill, 1997). The M182T substitution was shown to restore protein expression levels of TEM-1 L76N in *E. coli* and, importantly, restored expression of other destabilizing mutations in TEM-1. Based on these results, it was suggested that in natural ESBLs, M182T acts as a suppressor of folding and stability defects associated with substitutions that increase catalysis of extended-spectrum cephalosporins or cause inhibitor resistance (Huang and Palzkill, 1997). It was named a “global suppressor” based on similar properties to intragenic second-site stabilizing mutations that had previously been observed in staphylococcus nuclease that were named global suppressors (Shortle and Lin, 1985).

It was later shown that the TEM-1 L76N substitution results in misfolding leading to aggregation and proteolysis of the enzyme in the periplasmic space of *E. coli* (Sideraki et al., 2001). The addition of the M182T substitution restored the accumulation of active, folded enzyme in the periplasm and suppressed the formation of aggregates. It was also shown that, in guanidinium hydrochloride denaturation experiments, M182T did not act on the final stability of the L76N enzyme (Sideraki et al., 2001). Subsequently, it has been shown using circular dichroism (CD) measurements at increasing temperatures that M182T does increase the thermodynamic stability of the wild-type TEM-1 enzyme (Wang et al., 2002). In addition, it was shown using the CD assay that TEM mutations that increase oxyimino-cephalosporin hydrolysis such as R164S and G238S, also decrease the thermodynamic stability of the enzyme, i.e., there is a stability-function trade-off (Wang et al., 2002). Previous studies had also shown that R164S and G238S have reduced stability relative to wild-type TEM-1 (Raquet et al., 1995). Adding the M182T substitution to the G238S enzyme results in increased thermodynamic stability suggesting that M182T compensates for TEM ESBL substitutions that trade off protein stability for improved oxyimino-cephalosporin hydrolysis (Wang et al., 2002).

Consistent with the results of Wang et al. (2002), Knies et al. recently showed that M182T increases the thermostability of wild type TEM-1, that the G238S substitution results in lower thermostability of TEM-1, and that the M182T substitution restores stability to the M182T/G238S mutant (Knies et al., 2017). These authors note, however, that the increased thermostability does not correlate with increased cefotaxime MICs. They go on to suggest that the effects of the mutations on other factors may be important, such as folding/misfolding or kinetic stability (Knies et al., 2017). Studies on *in vivo* (in periplasm) folding kinetics and propensity for misfolded products of TEM ESBL mutants such as G238S and the influence of mutations including M182T on such folding will be of interest to further address the *in vivo* effect of ESBL substitutions and the role of global suppressor substitutions.

There have been multiple proposals for the structural basis by which M182T increases enzyme thermostability. Farzaneh et al. proposed that the threonine at position 182 results in a new hydrogen bond between residue 182 and the main chain carbonyl group of residue 64 (Farzaneh et al., 1996). This would provide an additional link between the α and αβ domains of β-lactamase and possibly increase stability. Alternatively, it has been shown that Thr182 acts as an N-cap residue for the residue 183-195 α-helix by forming and additional hydrogen bond to Ala185 that would be expected to increase stability (Minasov et al., 2002).

Finally, it is also noteworthy that several groups have now shown that there are, in fact, many substitutions in TEM-1 that increase the stability of the enzyme including V31R, I47V, F60Y, P62S, G78A, V80I, S82H, G92D, R120G, E147G, H153R, M182T, A184V, T188I, L201P, I208M, A224V, E240H, R241H, I247V, T265M, R275Q, R275L, and N276D (Bershtein et al., 2008; Kather et al., 2008; Marciano et al., 2008; Brown et al., 2010; Deng et al., 2012). A number of these substitutions have been observed in TEM ESBL or inhibitor-resistant enzymes from clinical isolates including G92D, R120G, H153R, M182T, A184V, A224V, T265M, R275Q, and N276D (Brown et al., 2010). Therefore, suppression of folding and stability defects is likely achieved by several mutational pathways in natural variants.

## Multiple substitutions and epistasis in TEM ESBLs

TEM ESBLs from resistant clinical isolates contain 1–5 substitutions and multiply substituted enzymes are the rule with single substitutions being relatively rare (www.lahey.org/studies/webt.asp). Many ESBLs contain a core substitution of either R164S or G238S with additional substitutions such as E104K, M182T, A237T or E240K. Enzymes containing R164S are more often associated with high ceftazidime resistance while those with G238S are associated with high cefotaxime resistance. This is related to the fact, as discussed above, that the R164S substitution gives a large increase in *k*_cat_/*K*_M_ for ceftazidime relative to wild type TEM-1 and G238S gives a large increase in cefotaxime *k*_cat_/*K*_M_ relative to wild type. This categorization is an oversimplification, however, in that R164S does moderately increase cefotaxime hydrolysis and G238S moderately increases ceftazidime hydrolysis relative to wild-type TEM-1.

The addition of substitutions to R164S and G238S mutations results in increased oxyimino-cephalosporin hydrolysis. For example, the addition of E104K to R164S results in a 30-fold increase in *k*_cat_/*K*_M_ for ceftazidime and a 2.4-fold increase for cefotaxime hydrolysis relative to the R164S enzyme (Sowek et al., 1991). Addition of the E240K substitution to R164S results in a 7-fold increase in *k*_cat_/*K*_M_ for ceftazidime but only a modest 1.2-fold increase for cefotaxime hydrolysis (Sowek et al., 1991). Further, the addition of E104K to the G238S enzyme results in a 15-fold increase in *k*_cat_/*K*_M_ for ceftazidime and a 10-fold increase for cefotaxime hydrolysis relative to the G238S enzyme (Wang et al., 2002). The addition of E240K to the G238S enzyme results in a 37-fold increase in *k*_cat_/*K*_M_ for ceftazidime and a 2.6-fold increase for cefotaxime hydrolysis relative to G238S (Venkatachalam et al., 1994). Taken together, these findings show that the E104K and E240K substitutions increase *k*_cat_/*K*_M_ of either R164S or G238S for both ceftazidime and cefotaxime. However, the effect is more pronounced for ceftazidime. It has been noted by Sowek et al., the effect of E104K and E240K on ceftazidime hydrolysis may be due to favorable electrostatic interactions of lysine in either position with the carboxylate group found in the C7β side chain of ceftazidime (Sowek et al., 1991).

R164S or G238S mutations in combination with E104K or E240K generally result in additive effects on catalysis where the combination of two substitutions that each increase hydrolysis leads to even higher levels of hydrolysis in the double mutant. Additive combinations reveal that the two substitutions have independent effects on catalysis (Wells, 1990). However, not all substitutions associated with TEM ESBLs are additive. For example, the combination of E104K and E240K, each of which increases *k*_cat_/*K*_M_ for cefotaxime hydrolysis when introduced individually into the TEM-1 enzyme, results in an E104K/E240K double mutant that hydrolyzes cefotaxime with a *k*_cat_/*K*_M_ at the same level as the E104K substitution alone (Hart et al., 2016). Similarly, the addition of E104K to a R164S/E240K enzyme results in an E104K/R164S/E240K mutant that exhibits the same *k*_cat_/*K*_M_ value for cefotaxime hydrolysis as the R164S/E240K enzyme (Sowek et al., 1991). Therefore, in these examples, the presence of E240K negates the effect of E104K (and vice versa). The lack of additivity means that the two mutations interact, directly or indirectly, and that the interaction has a negative effect on hydrolysis.

Non-additive effects of mutations are termed epistasis (de Visser and Krug, 2014; Dellus-Gur et al., 2015). Epistasis is an important element of evolution because it determines what mutational pathways are accessible to natural selection (de Visser and Krug, 2014; Dellus-Gur et al., 2015). Indeed, these effects have been highlighted for a TEM mutant consisting of a promoter mutation (g4205a) as well as the amino acid substitutions A42G/E104K/M182T/G238S. This variant provides high-level cefotaxime resistance to *E. coli* (Stemmer, 1994; Orencia et al., 2001). Weinreich et al. showed that out of the 5! = 120 mutational pathways leading from wild type to the final mutant, 102 were selectively inaccessible paths in that some of the individual mutations do not increase cefotaxime resistance on all allelic backgrounds. Because some increase in cefotaxime resistance is required for selection of intermediate mutations, mutational pathways that include a step with no selection advantage will be dead-ends. Thus, negative epistasis excludes some of the possible pathways by which complex mutations may arise (Weinreich et al., 2006).

A well-studied example of epistasis in the TEM system from a structural and functional standpoint is the combination of R164S with G238S. The combination of R164S and G238S, each of which increase resistance toward cefotaxime and ceftazidime, results in a double mutant R164S/G238S with reduced resistance toward each of these (Giakkoupi et al., 2000). The *k*_cat_/*K*_M_ for cefotaxime hydrolysis by the R164S/G238S double mutant is the same as R164S and 20-fold lower than that for G238S, indicating negative epistasis between the substitutions (Dellus-Gur et al., 2015). In each case, the *k*_cat_/*K*_M_ for the double mutant is significantly smaller that the *k*_cat_/*K*_M_ expected if the two mutations were simply additive.

In order to understand the molecular basis of the observed epistasis, the structure of the R164S/G238S double mutant was determined by Dellus-Gur et al. in the context of other stabilizing substitutions that do not impact kinetic parameters as described above for G238S and R164S (Dellus-Gur et al., 2015). As noted above, the G238S substitution induced two dominant conformations of the G238-loop while the R164S substitution induced an ensemble of conformations of the omega loop. The R164S/G238S double mutant exhibited a wider ensemble of conformations than the single mutants (Dellus-Gur et al., 2015). In addition, a non-native interaction between residues 171 and 240 is present which results in a dominant, large change in the position of the catalytically important residue Asn170. It was suggested that the entropic cost of the substrate selecting from the many conformations, in addition to the unfavorable position of Asn170 results in the low *k*_cat_ for cefotaxime hydrolysis by the double mutant, thereby accounting for the negative epistatic effect of introducing the second mutation (Dellus-Gur et al., 2015). Based on the results, the authors suggest there is a delicate balance between the adaptive benefit of increased structural freedom to allow oxyimino-cephalosporin access to the active site and the cost of diluting the catalytically optimal conformation among many non-productive conformations that decreases catalytic efficiency (Dellus-Gur et al., 2015). This study also provides evidence for the importance of conformational ensembles or sub-states in enzyme action and evolution. The coexistence of multiple sub-states has been referred to as “floppiness” and is recognized as an important component of enzyme catalytic efficiency and evolution (Tokuriki and Tawfik, 2009a; Bar-Even et al., 2015).

## Computational studies of conformational heterogeneity of TEM ESBLs

Computational studies also support a role for conformational flexibility in the evolution of altered specificity in the TEM-1 enzyme. It has been shown through docking and molecular dynamics that the G238S and E104K substitutions induce changes in the conformation of the omega loop as well as regions consisting of residues 86–118, 213–229, and 267–271 upon binding cefotaxime (Singh and Dominy, 2012). In addition, a study has demonstrated flexibility in TEM-1 and greater flexibility in ancestral β-lactamases that exhibit broader substrate specificity, thereby linking flexibility with broad specificity (Zou et al., 2015). Further, hidden allosteric sites have been discovered in the TEM enzyme (Horn and Shoichet, 2004; Bowman et al., 2015). These sites are binding pockets that are not present in the crystal structure but become available as the protein structure fluctuates in solution. Such a site was discovered via structure determination of a small molecule inhibitor that was found bound in a pocket 16 Å from the active site that is not apparent in the apoenzyme (Horn and Shoichet, 2004). The conformational changes associated with small molecule binding in the cryptic pocket were communicated to the active site resulting in movement of a key active site residue thereby inhibiting the enzyme (Horn and Shoichet, 2004). Hidden allosteric sites that are sampled among TEM-1 conformations have also been discovered computationally and thiol labeling experiments support their presence in TEM-1 (Bowman and Geissler, 2012; Bowman et al., 2015). Taken together, these studies support a role for conformational heterogeneity in β-lactamases and its potential impact on catalysis via communication with the active site.

Recently, molecular dynamics simulations and Markov State Models (MSMs) were used to examine the mechanism of action of the TEM-1 wild type, E104K, and G238S substitutions on cefotaxime hydrolysis (Hart et al., 2016). The authors showed that docking of cefotaxime into static TEM ESBL structures shows a poor correlation with *k*_cat_/*K*_M_ of the variants, suggesting the static structures do not contain sufficient information to understand the function of the enzymes. The correlation was improved using “Boltzman docking” where MSMs based on molecular dynamics simulations were weighted to the contribution of each state by its equilibrium probability (Hart et al., 2016). The results suggested that consideration of enzyme conformational sub-states rather than a single structure provides more relevant information on TEM ESBL activity against cefotaxime. Next, the authors used MSMs constructed based on molecular dynamics of wild type TEM-1 and the E104K/G238S enzyme that hydrolyzes cefotaxime >1,000-fold faster than wild type and identified states that are more populated by E104K/G238S than by wild type. Interestingly, it was found that the “cefotaximase states” resembled the wild-type structure while the omega loop in the wild type undergoes substantial rearrangements (Hart et al., 2016). This suggests that E104K/G238S hydrolyzes cefotaxime more efficiently than wild type because the omega loop is actually more constrained in a conformation that will accommodate cefotaxime in the double mutant while the wild-type TEM-1 samples many non-productive conformations. Support for this model was obtained by chemical footprinting and the construction of mutations predicted to preferentially occupy active conformational states (Hart et al., 2016). These findings suggest that, contrary to the view that the active site needs to become more open to accommodate cefotaxime, for productive catalysis it needs to be more constrained to limit non-productive sub-states (Hart et al., 2016). Examination of the most populated states of E104K/G238S cefotaximase indicated the serine at position 238 and lysine at position 104 act to pin down the omega loop to limit non-productive conformations (Hart et al., 2016). These results again highlight the importance of conformational sub-states and provide a different view from the idea that the expansion of the TEM-1 active site by ESBL mutations to accommodate oxyimino-cephalosporins is the causative basis of enhanced catalytic efficiency.

## CTX-M β-lactamase variants and ceftazidime hydrolysis

CTX-M β-lactamases are class A enzymes that are characterized by the ability to efficiently hydrolyze cefotaxime (Bonnet, 2004). These enzymes have spread globally to become the most widespread ESBLs in Gram-negative bacteria (Cantón et al., 2012). The CTX-M ESBLs are divided into five clusters based on amino acid sequence homology including CTX-M-1, CTX-M-2, CTX-M-8, CTX-M-9, and CTX-M-25 with the names based on the prominent member of each subgroup (D'Andrea et al., 2013). The subgroups differ from one another by >10% amino acid sequence divergence and each subgroup contains a number of variants that differ from one another by <5% sequence divergence (D'Andrea et al., 2013). The CTX-M enzymes are ~35% identical to TEM-1 β-lactamase.

The Toho-1 (CTX-M-44), CTX-M-9, and CTX-M-14 enzymes have been the most intensively studied in terms of structure and mechanism (Shimamura et al., 2002; Chen et al., 2005; Delmas et al., 2010). Toho-1 is in the CTX-M-2 subfamily while CTX-M-9 and −14 are in the CTX-M-9 subfamily and differ only in a V231A substitution in CTX-M-9 relative to CTX-M-14 (Chen et al., 2005; D'Andrea et al., 2013). In addition, CTX-M-9 and CTX-M-14 exhibit similar kinetic parameters for β-lactam substrate hydrolysis (Chen et al., 2005). Therefore, structure and function conclusions from studies of one of these enzymes are likely to apply to the others.

**Figure 6:**
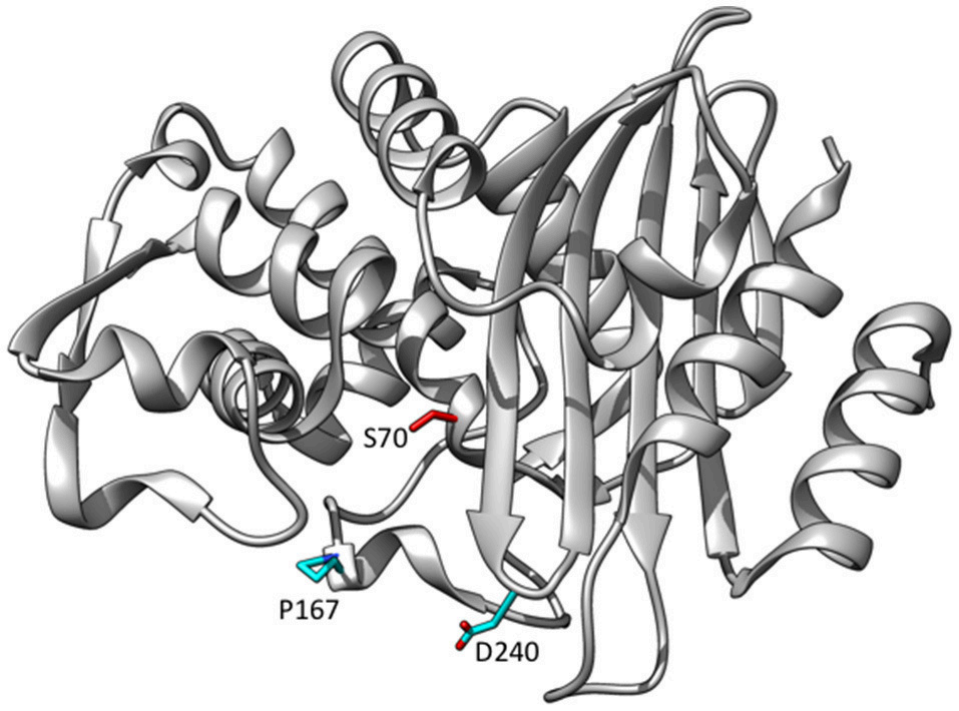
Ribbon diagram of CTX-M-14 β-lactamase showing the positions of residues Pro167 and Asp240 that are substituted in variants able to hydrolyze ceftazidime (cyan). The catalytic residue Ser70 is shown in red. (PDB ID: 1YLT).

The focus here will be on CTX-M-14 and the role of amino acid substitutions found in variants that increase ceftazidime hydrolysis. In contrast to TEM-1, the CTX-M enzymes are excellent catalysts for the hydrolysis of cefotaxime (Bonnet, 2004). With regard to CTX-M-14, *k*_cat_ is in the range of 200–400 s^−1^ and *K*_M_ ~100 μM while *k*_cat_/*K*_M_ is ~3.0 × 10^6^ M^−1^s^−1^ (Chen et al., 2005; Adamski et al., 2015; Patel et al., 2015). Thus, the *k*_cat_/*K*_M_ value for cefotaxime hydrolysis by CTX-M-14 is about 1,500-fold higher than that for TEM-1. Structural studies of CTX-M-14 and CTX-M-9 apoenzymes reveal that, contrary to the expectation that a wider active site would better accommodate cefotaxime, the active site is narrower than that observed for TEM-1 (Chen et al., 2005; Delmas et al., 2010). X-ray structures of CTX-M-14 and CTX-M-9 in the presence of cefotaxime show that the active site expands significantly with cefotaxime bound in the active site (Delmas et al., 2010; Adamski et al., 2015). These structures were solved using an S70G substitution of the nucleophile for acylation in order to trap the unhydrolyzed substrate (Delmas et al., 2010). The expansion is accompanied by the breaking of a hydrogen bond between the main chain carbonyl oxygen of Asn170 and the main chain NH of Asp240 that connects to omega loop the β-3 strand (Delmas et al., 2010) (Figure 6). Note that this is a similar region of the active site as that of TEM-1 where the G238S substitution increases the hydrolysis of oxyimino-cephalosporins and the proposed effect of increasing the size of the active site is similar.

Although CTX-M enzymes rapidly hydrolyze cefotaxime, the related oxyimino-cephalosporin ceftazidime is poorly hydrolyzed (Bonnet, 2004). Determinations of kinetic parameters for CTX-M-14 for ceftazidime reveal high *K*_M_ values of >600 μM, *k*_cat_ values <5 s^−1^ and a *k*_cat_/*K*_M_ of 1–5 × 10^3^ M^−1^s^−1^ (Chen et al., 2005; Patel et al., 2015). This *k*_cat_/*K*_M_ value is ~1,000-fold less than *k*_cat_/*K*_M_ for cefotaxime hydrolysis by CTX-M-14. Note, however, that this *k*_cat_/*K*_M_ value for ceftazidime is still ~100-fold higher than that observed for TEM-1 indicating CTX-M is a significantly better catalyst for this substrate than TEM-1. The difference in *k*_cat_/*K*_M_ for ceftotaxime vs. ceftazidime by CTX-M enzymes is due to the extra bulk of the C7β side chain of ceftazidime that contains a carboxypropyl group replacing the methyl group found in cefotaxime. This leads to steric clashes in binding ceftazidime in the CTX-M active site (Delmas et al., 2008).

## CTX-M D240G substitution

As noted, the CTX-M enzymes hydrolyze ceftazidime poorly compared to cefotaxime. In the last 15 years, however, variants have emerged that are able to hydrolyze ceftazidime (Bonnet, 2004; Cantón et al., 2012; D'Andrea et al., 2013). In particular, the D240G and P167S substitutions occur individually in multiple CTX-M subgroups where they enhance ceftazidime hydrolysis (Bonnet et al., 2001, 2003; Cartelle et al., 2004; Kimura et al., 2004; Ishii et al., 2007). For example, the D240G substitution has been identified in the CTX-M-1, −2, −9, and −25 subfamilies while the P167S substitution has been identified in variants belonging to the CTX-M-1 and−9 subfamilies (D'Andrea et al., 2013). CTX-M variants containing D240G or P176S from clinical isolates are associated with increased MIC values for ceftazidime and the introduction of the substitutions into CTX-M-14 results in increased ceftazidime MICs for *E. coli* containing the mutants vs. the wild-type CTX-M-14 (Patel et al., 2015).

The D240G substitution, when introduced into CTX-M-14 or CTX-M-9, results in a 10-fold increase in *k*_cat_/*K*_M_ for ceftazidime hydrolysis (Bonnet et al., 2003; Chen et al., 2005; Patel et al., 2015). Residue 240 is at the end of the β3-strand that flanks the active site and also contains Thr235 and Ser237 that make direct interactions with the C4 carboxylate group of cephalosporins (Delmas et al., 2010; Adamski et al., 2015; Figure 6). The X-ray crystal structure of CTX-M-14 containing the D240G substitution (CTX-M-27) has been determined, and anisotropic B-factor analysis demonstrated increased flexibility of the β3- strand that forms the side of the active site (Chen et al., 2005). This increase in flexibility is thought to expand its substrate profile by allowing access to the bulkier ceftazidime molecule (Chen et al., 2005).

The effect of the D240G substitution was further investigated by determining the structure of CTX-M-9 with the D240G substitution in complex with a glycylboronic acid containing the C7β oximino side chain of ceftazidime which forms a covalent adduct with Ser70 and places the ceftazidime side chain in a similar position as cefotaxime in the Toho-1 E166A-cefotaxime structure (Shimamura et al., 2002; Delmas et al., 2008). A comparison of the positioning of the ceftazidime side chain in the D240G mutant to its position in the wild type CTX-M-9 revealed that the aminothiazole ring was positioned deeper into the active site, more resembling the position of the aminothiazole ring in cefotaxime structures (Delmas et al., 2008). This positioning, in turn, led to more optimal contacts of the NH of the amide group of the ceftazidime side chain with the Ser237 main chain oxygen. Molecular dynamics simulations of ceftazidime acyl-enzyme structures of CTX-M-9 and the D240G mutant based on the ceftazidime-like boronic acid structures also suggested more optimal contacts of Thr235 with the C4 carboxylate of ceftazidime. A new hydrogen bond is observed between the amino group of the aminothiazole ring and the Pro167 backbone oxygen, along with an improved Ser237 main chain O interaction with the amide group (Delmas et al., 2008). Thus, the D240G substitution, via deeper positioning of the aminothiazole ring, appears to improve interactions with ceftazidime with multiple active site groups (Delmas et al., 2008).

## CTX-M P167S substitution

The CTX-M P167S substitution, when introduced into CTX-M-14, results in a 10-fold increase in *k*_cat_/*K*_M_ for ceftazidime hydrolysis, similar to that observed for D240G (Patel et al., 2015). Pro167 resides on the omega loop and the peptide bond between Glu166 and Pro167 is in the *cis* configuration (Figure 6). The *cis* peptide bond contributes strongly to the conformation of the omega loop and particularly the positioning of Asn170 (Patel et al., 2017). Pro167 is largely conserved among class A β-lactamases, including TEM-1, and is a *cis*-proline in these enzymes (Philippon et al., 2016).

Molecular dynamics simulations based on the structure of the Toho-1 (CTX-M-44) enzyme have been performed to assess the effect of the P167S substitution (Kimura et al., 2004). The results suggested that in the P167S enzyme, the aminothiazole ring is displaced to prevent steric clash with Ser167 causing the C4 carboxylate of ceftazidime to hydrogen bond to Ser130 and Ser237 and enhancing hydrolysis (Kimura et al., 2004).

**Figure 7:**
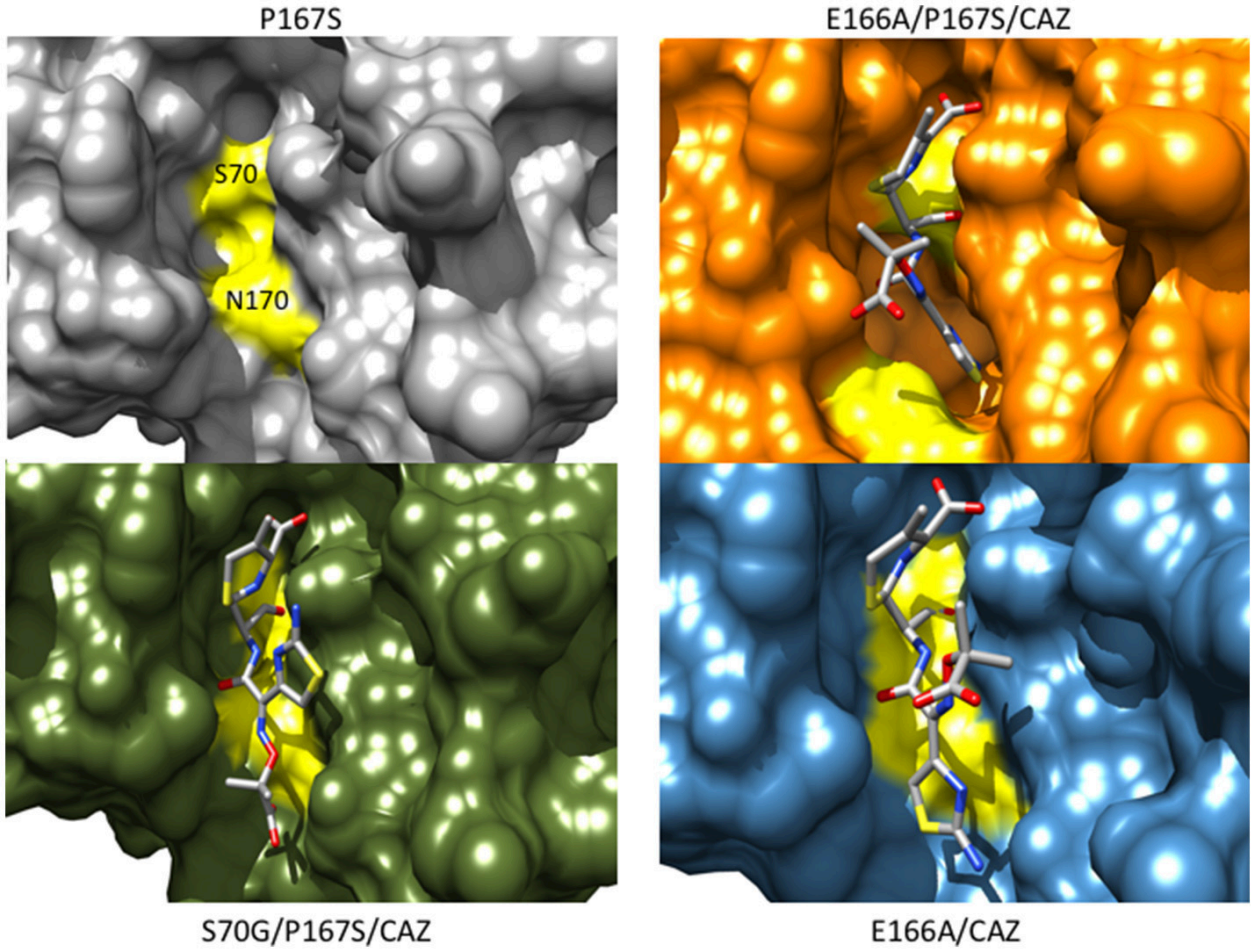
Protein surface representations of the CTX-M-14 P167S (gray) (PDB ID:5TWD), S70G/P167S with ceftazidime (green) (5TWE), E166A/P167S with acylated ceftazidime (orange) (5TW6) and E166A with acylated ceftazidime (blue) (5U53) are shown. The positions of Ser70 and Asn170 on the CTX-M structure are shown in yellow illustrating the movement of the omega loop in the E166A/P167S/CAZ acyl-enzyme separates Ser70 and Asn170 to create space in the active site to accommodate ceftazidime.

**Figure 8:**
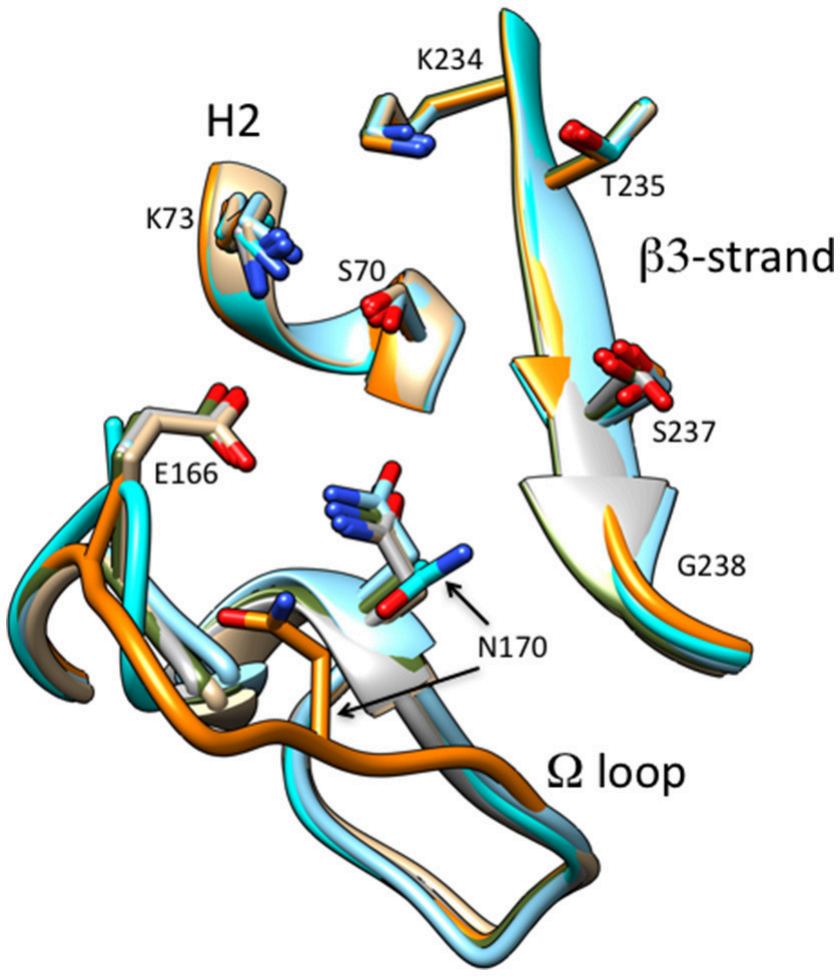
Schematic illustration of the omega loop region as well as the β3-strand and H2 helix of the active site of CTX-M-14 wt (tan) (PDB ID:1YLT) in comparison P167S (gray) (PDB ID:5TWD), S70G/P167S with ceftazidime (green) (5TWE), E166A/P167S with acylated ceftazidime (orange) (5TW6), E166A/P167S apoenzyme (cyan) (5VTH) and E166A with acylated ceftazidime (blue) (5U53). The omega loop in E166A/P167S with acylated ceftazidime undergoes a large conformational change moving Asn170 out of the active site. The ceftazidime molecule is omitted for clarity.

The CTX-M-14 β-lactamase has also been used as a model system to examine the structural changes caused by the P167S substitution that are associated with increased ceftazidime hydrolysis. A number of X-ray structures of CTX-M-14 P167S were solved that enabled an evaluation of the changes in structure between the apoenzyme, the initial enzyme complex with ceftazidime, and the formation of the ceftazidime-acyl enzyme (Patel et al., 2017). The S70G mutation was introduced to P167S to prevent acyl-enzyme formation and capture the structure of the enzyme-substrate complex and the E166A mutation was introduced to P167S to prevent deacylation of the acyl-enzyme to observe the acyl-enzyme structure. It was observed that the P167S apoenzyme closely resembled CTX-M-14 and the peptide bond preceding Ser167 was *cis*, despite a non-prolyl *cis* peptide bond being very energetically unfavorable (Jorgensen and Gao, 1988; Patel et al., 2017). The S70G/P167S structure in complex with ceftazidime revealed that the substrate bound with the C8 carbonyl oxygen in the oxyanion hole, as expected for a productive complex. The C4 carboxylate was bound by Thr235 but the C7β side chain was more solvent exposed due to steric constraints with the omega loop (Figure 7). The omega loop retained the conformation observed for the wild-type CTX-M-14 and P167S enzymes, and the peptide bond preceding Ser167 was also *cis* (Patel et al., 2017; Figure 8). The addition of E166A to the P167S enzyme to create E166A/P167S also showed the omega loop in a closed conformation in the apoenzyme structure, similar to the wild type and P167S apoenzymes (Figure 8). However, the peptide bond preceding Ser167 was *trans*. The addition of ceftazidime to E166A/P167S to form an acyl-enzyme resulted in a *trans* peptide bond preceding Ser167 and a large conformational change of the omega loop with Asn170 moving 4.2 Å out of the active site to create a pocket to accommodate the ceftazidime side chain (Patel et al., 2017; Figures 7, 8). This resulted in the aminothiazole ring sinking deeply into the active site in a buried position (Figure 7). These results suggested that both the Ser167 substitution and the presence of acylated ceftazidime are required for the conformational change. Finally, in order to show that the conformational change was due to the P167S substitution and not the E166A substitution that was used to trap the acyl-enzyme, the structure of E166A in complex with ceftazidime was determined. This showed the acyl-ceftazidime in the active site with Pro167 in the *cis* configuration and the omega loop in the closed conformation indicating the P167S substitution is required for the conformational change (Figures 7, 8). Therefore, the P167S substitution and the presence of acylated ceftazidime were both necessary to drive the structure toward a *trans* peptide bond at residue 167 and to induce conformational change of the omega loop (Patel et al., 2017).

It is interesting to note that the structure of the CTX-M-9 D140G enzyme in complex with the ceftazidime-like boronic acid inhibitor and the CTX-M-14 E166A/P167S enzyme with acylated ceftazidime show the aminothiazole ring sinking to a deeper position in the active site relative to CTX-M-9 or CTX-M-14, respectively (Delmas et al., 2008; Patel et al., 2017). This suggests the P167S and D240G substitutions utilize different paths to a broadly similar end result—altered positioning of the aminothiazole ring and better contacts of substrate with the enzyme. In addition, the TEM-64 (E104K/R164S/M182T) ESBL enzyme with increased activity toward ceftazidime also displays a similar conformational change of the omega loop when complexed to a boronic acid inhibitor (Wang et al., 2002). Finally, a TEM-1 triple mutant (W165Y/E166Y/P167G) selected from a random mutagenesis experiment for increased hydrolysis of ceftazidime displays a large conformational change of the omega loop creating space to accommodate ceftazidime (Stojanoski et al., 2015). These studies suggest that enlargement of the active site via movement of the omega loop, which results in a shift of the Asn170 residue, is an important mechanism by which mutations can lead to hydrolysis of the bulky ceftazidime molecule in class A β-lactamases.

## Stabilizing substitutions in CTX-M natural variants

It has been demonstrated that the P167S and D240G substitutions both destabilize the CTX-M enzyme (Chen et al., 2005; Patel et al., 2015). As indicated above, the CTX-M family consists of a large number of variants containing amino acid substitutions (D'Andrea et al., 2013). Many of the substitutions are located outside of the active site and their effect on CTX-M structure and function is largely unknown. The A77V substitution is found in multiple subfamilies including the CTX-M-1, CTX-M-9, and CTX-M-25 groups (Patel et al., 2015). In addition, A77V has been found associated with either the P167S or D240G substitutions in each of these subfamilies. Using the CTX-M-14 model system, addition of the A77V substitution to either a P167S or D240G enzyme results in enhanced steady-state protein expression levels in *E. coli* relative to the P167S or D240G single mutants (Patel et al., 2015). In addition, the A77V/P167S and A77V/D240G enzymes exhibit increased thermal stability *in vitro* compared to the P167S and D240G enzymes (Patel et al., 2015). Based on these results, A77V has been suggested to be a global suppressor for CTX-M, analogous to M182T for TEM-1. Given the large number of substitutions among CTX-M variants, it is likely that other global suppressors are present in the family of clinical variants.

## KPC β-lactamase variants and ceftazidime hydrolysis

KPC-2 β-lactamase is a class A enzyme that hydrolyzes a broad range of β-lactam antibiotics including pencillins, cephalosporins and carbapenems. This enzyme also efficiently hydrolyzes cefotaxime but only poorly hydrolyzes ceftazidime. The value for *k*_cat_/*K*_M_ for cefotaxime hydrolysis is ~3.0 × 10^5^ M^−1^s^−1^ (Yigit et al., 2003; Levitt et al., 2012) while *k*_cat_/*K*_M_ for ceftazidime is ~1.0 × 10^3^ M^−1^s^−1^ (Mehta et al., 2015). By comparison, *k*_cat_/*K*_M_ for ceftazidime for KPC-2 is 25-fold higher than that for TEM-1 and similar to the *k*_cat_/*K*_M_ value for CTX-M-14. KPC-2 is an important clinical problem due to its broad substrate profile and wide distribution in enteric bacteria (Nordmann et al., 2009). The problem is compounded by the evolution of KPC variants that with increased ceftazidime hydrolysis rates and resistance (Mehta et al., 2015; Naas et al., 2016). Over 20 variants of KPC have been identified that contain a range of amino acid substitutions (Naas et al., 2016).

**Figure 9:**
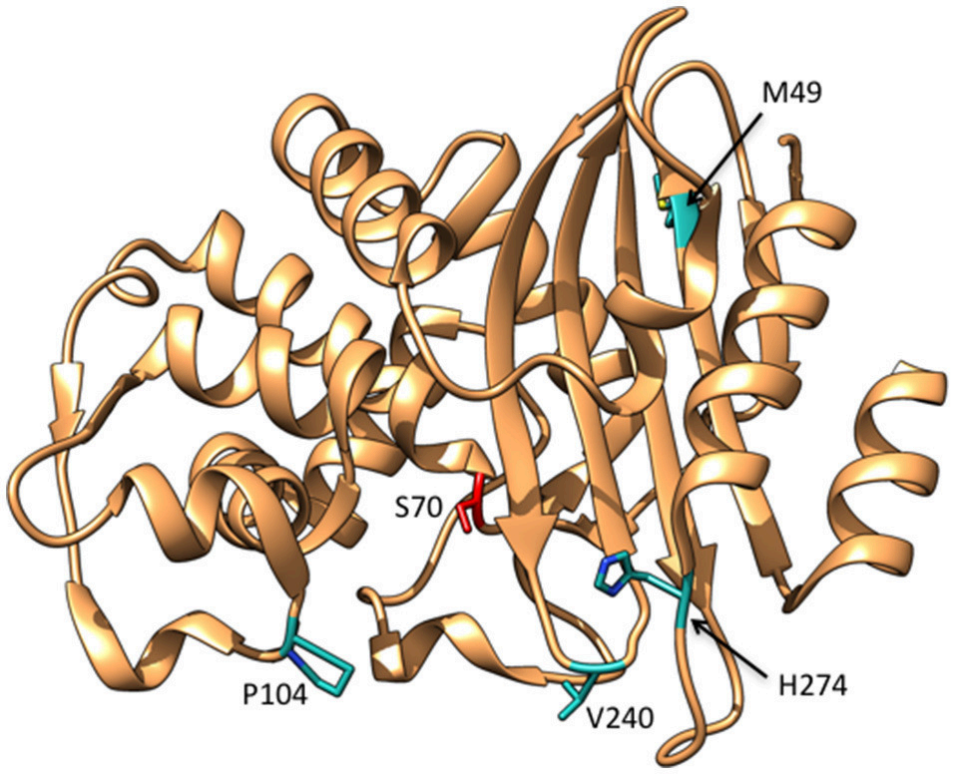
Ribbon diagram of KPC-2 β-lactamase showing the positions of residues Met49, Pro104, Val240, and His274 that are substituted in variants able to hydrolyze ceftazidime (cyan). The catalytic residue Ser70 is shown in red. (PDB ID:2OV5).

A detailed study of the KPC-3 to KPC-11 variants showed that these enzymes exhibit increased *k*_cat_/*K*_M_ values for ceftazidime hydrolysis compared to KPC-2 (Mehta et al., 2015). Amino acid substitutions found among these enzymes include M49I, P104R, P104L, V240G, V240A, and H274Y (Figure 9). The P104R single substitution was found to increase *k*_cat_/*K*_M_ for ceftazidime by 10-fold compared to KPC-2 while P104L exhibited a more modest 2.5-fold increase (Mehta et al., 2015). The V240G substitution increased *k*_cat_/*K*_M_ for ceftazidime by 5-fold and H274Y resulted in a 10-fold increase. Double mutants containing combinations of the single substitutions exhibited further increases in *k*_cat_/*K*_M_. For example, the V240G/H274Y, P104R/V240G, and P104R/H274Y enzymes displayed 40-, 50-, and 75-fold increases in *k*_cat_/*K*_M_, respectively, for ceftazidime relative to KPC-2 (Mehta et al., 2015). In contrast, the M49I/H274Y enzyme exhibited similar catalytic efficiency as the H274Y enzyme, suggesting that M49I does not contribute to ceftazidime hydrolysis; however, the M49I substitution alone was not studied. Further, it was shown that the P104R, V240G, and H274Y substitutions act additively rather than cooperatively, i.e., there is no epistasis, when combined into the double mutants. This indicates that the substitutions act independently and do not influence each other's function when present in the double mutants. This would also suggest that the order in which the mutations occur to form the double mutant is not important (Mehta et al., 2015).

There is currently no structural information on the variants so there is limited understanding of their effect on KPC or the mechanism by which they alter specificity. However, molecular modeling of the substitutions onto the KPC-2 structure and computational docking of ceftazidime suggested that the P104R and H274Y substitutions could create new hydrogen bonding interactions with the C7β-oxyimino side chain of ceftazidime (Mehta et al., 2015). Note that the P104R substitution is in the same region of the proteins as the E104K substitution that commonly occurs in TEM ESBL variants. In addition, the V240G substitution is analogous to the CTX-M D240G substitution and could act by a similar mechanism of increasing flexibility of the β3 strand and allowing the aminothiazole ring of ceftazidime to sink deeper into the active site upon binding (Chen et al., 2005; Delmas et al., 2008). Finally, the H274Y substitution appears to be unique to the KPC variants.

As noted above, several substitutions in TEM and CTX-M that increase oxyimino-cephalosporin hydrolysis also decrease stability (Wang et al., 2002; Chen et al., 2005; Patel et al., 2015; Knies et al., 2017). A similar pattern was observed with the substitutions found in the KPC-3 to KPC-11 variants. There was a strong inverse correlation between *k*_cat_/*K*_M_ for ceftazidime hydrolysis and the T_m_ of the enzymes (Mehta et al., 2015). However, the T_m_ for the wild type KPC-2 (66°C) is significantly higher than that for TEM or CTX-M and even the most unstable KPC variant is more stable than wild-type TEM-1 (Mehta et al., 2015). Therefore, the high stability of KPC-2 may serve as a buffer to allow the accumulation of substitutions without causing unfolding of the enzyme. This type of stability buffer has been noted previously as a mechanism to enhance protein evolvability (Bloom and Arnold, 2009; Tokuriki and Tawfik, 2009b).

## Conclusions

The use of oxyimino-cephalosporins in the clinical setting has led to the evolution of variants of TEM-1, CTX-M, and KPC enzymes that can hydrolyze these drugs. The TEM-1 enzyme does not efficiently hydrolyze either cefotaxime or ceftazidime but there are now many variants that exhibit increased hydrolysis of these drugs. Initial isolates of CTX-M and KPC enzymes hydrolyze cefotaxime but not ceftazidime; however, variants now exist that hydrolyze ceftazidime. For TEM-1, recent studies have pointed to the importance of conformational heterogeneity of variants with ESBL substitutions. Rather than adopting a single, stable conformation, some variants exist in multiple sub-states of conformations that contain active conformers that can accommodate oxyimino-cephalosporins. One study also indicates that sampling of non-productive sub-states underlies the failure of mutant combinations such as R164S/G238S and, in another study, wild-type TEM-1 itself, to hydrolyze oxyimino-cephalosporins. Studies with TEM-1 have also indicated that several ESBL substitutions increase catalytic activity but are associated with a cost in stability. Global suppressor mutations then occur to compensate for the stability loss. It will be of interest to examine the folding, aggregation and stability effects of ESBL mutations and the compensatory effects of suppressors *in vivo* in the periplasm. The substitutions associated with ceftazidime hydrolysis in CTX-M are different than those observed in TEM, but act through conformational changes in similar regions of the active site as TEM-1. KPC variants are in analogous positions as some TEM and CTX-M substitutions and may act similarly. Conformational heterogeneity and the existence of sub-states have not been examined for CTX-M or KPC variants but may also be an important contributor to the function of these ESBL enzymes.

## Author contributions

The author confirms being the sole contributor of this work and approved it for publication.

### Conflict of interest statement

The author declares that the research was conducted in the absence of any commercial or financial relationships that could be construed as a potential conflict of interest.
